# Interleukin-20 is involved in dry eye disease and is a potential therapeutic target

**DOI:** 10.1186/s12929-022-00821-2

**Published:** 2022-06-09

**Authors:** Hsiao-Hsuan Wang, Wei-Yu Chen, Yi-Hsun Huang, Sheng-Min Hsu, Yeou-Ping Tsao, Yu-Hsiang Hsu, Ming-Shi Chang

**Affiliations:** 1 Institute of Basic Medical Sciences, College of Medicine, National Cheng Kung University, Tainan, Taiwan; 2 Department of Biochemistry and Molecular Biology, College of Medicine, National Cheng Kung University, Tainan, Taiwan; 3 Institute of Clinical Medicine, College of Medicine, National Cheng Kung University, Tainan, Taiwan; 4 Department of Ophthalmology, National Cheng Kung University Hospital, College of Medicine, National Cheng Kung University, Tainan, Taiwan; 5 Department of Medical Research, Mackay Memorial Hospital, Taipei, Taiwan; 6 Department of Ophthalmology, Mackay Memorial Hospital, Taipei, Taiwan; 7 Research Center of Clinical Medicine, National Cheng Kung University Hospital, Tainan, Taiwan

**Keywords:** Dry eye disease, Interleukin-20 (IL-20), Inflammation, Hyperosmotic stress

## Abstract

**Background:**

Dry eye disease (DED) is a common disease in ophthalmology, affecting millions of people worldwide. Recent studies have shown that inflammation is the core mechanism of DED. IL-20 is a proinflammatory cytokine involved in various inflammatory diseases. Therefore, we aimed to explore the role of this cytokine in the pathogenesis of DED and evaluate the therapeutic potential of the anti-IL-20 monoclonal antibody (mAb) 7E for DED treatment.

**Methods:**

Clinical tear samples from patients with DED and non-DED controls were collected and their IL-20 protein levels were determined. We established three DED animal models to explore the role of IL-20 and the efficacy of IL-20 antibody in DED. Benzalkonium chloride (BAC)-induced over-evaporative DED, extra-orbital lacrimal gland excision (LGE)-induced aqueous tear-deficient DED, and desiccating stress (DS)-induced combined over-evaporative and aqueous tear-deficient DED animal models were established to investigate the role of IL-20. The anti-IL-20 antibody 7E was established to neutralize IL-20 activity. The effects of IL-20 or 7E on human corneal epithelial cells and macrophages under hyperosmotic stress were analyzed. 7E was topically applied to eyes to evaluate the therapeutic effects in the DED animal models.

**Results:**

IL-20 was significantly upregulated in the tears of patients with DED and in the tears and corneas of DED animal models. Under hyperosmotic stress, IL-20 expression was induced via NFAT5 activation in corneal epithelial cells. 7E suppressed hyperosmotic stress-induced activation of macrophages. IL-20 induced cell death in corneal epithelial cells and 7E protected cells from hyperosmotic stress-induced cell death. Blocking IL-20 signaling with 7E protected mice from BAC-induced, LGE-induced, and DS-induced DED by reducing DED symptoms and inhibiting inflammatory responses, macrophage infiltration, apoptosis, and Th17 populations in the conjunctiva and draining lymph nodes.

**Conclusions:**

Our results demonstrated the functions of IL-20 in DED and presented a potential therapeutic option for this condition.

**Graphical Abstract:**

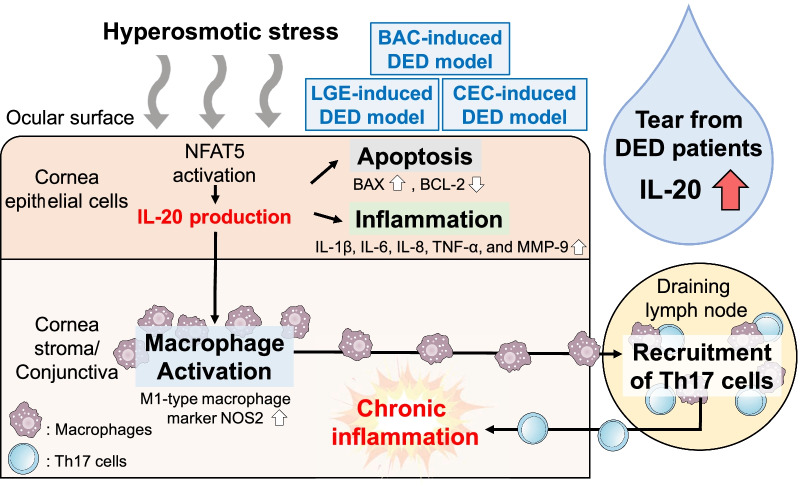

**Supplementary Information:**

The online version contains supplementary material available at 10.1186/s12929-022-00821-2.

## Background

Dry eye disease (DED) is the most common disease in ophthalmology, affecting millions of people worldwide. Currently, the world’s population is becoming an aging society, and DED may become a serious problem [1]. DED is characterized by a prolonged dry state of the cornea and conjunctiva of the outer layer of the eye, causing the eyes of patients to feel dry, red, itchy, and sensitive to external stimuli. The etiology and pathogenesis of DED are complex and multifactorial and may involve autoimmune diseases, contact lens use, hormonal changes, chronic inflammation, environmental factors, and infection. Patients with severe DED may even present with excessive eye watering, burning sensation, thick secretions, and blurred vision. Clinically, DED can be divided into two main types, aqueous tear-deficient dry eye and evaporative dry eye [2]. Aqueous tear-deficient dry eye can be subdivided into two categories, Sjögren’s dry eye type and non-Sjögren’s dry eye type. Evaporative dry eye has been subdivided to distinguish between those eyelid and ocular surface conditions that depend on intrinsic causes and those that arise from extrinsic influences. Studies have shown that many patients have both aqueous tear-deficient and evaporative dry eye, and both types share the common feature of inflammation [3]. It is essential to understand the pathogenesis and molecular mechanism of DED to facilitate drug development. Currently, no available drugs can effectively cure DED, and many cause negative side effects. Therefore, there is an urgent need to understand the pathogenesis of DED and to develop novel therapeutic strategies for DED treatment.

The cornea functions to protect the eyes from dust and bacteria and to focus vision. Normally, eyes are protected by the tear film outside the cornea. The tear film is mainly composed of three protein components, including the mucin layer (secreted by goblet cells on the conjunctiva), the aqueous layer (secreted by the lacrimal gland), and the lipid layer (secreted by the meibomian gland). If any layer displays insufficient secretion or excessive evapotranspiration, corneal epithelial cells become damaged under hyperosmotic conditions. In the long term, corneal epithelial cells secrete several proinflammatory cytokines, which in turn activate immune cells, causing continuous inflammation on the surface of the cornea. The inflammatory reaction even spreads to the goblet cell-rich conjunctiva, causing a loss of goblet cells and mucin [4]. The major mucin content in the conjunctiva, MUC5AC, is reduced in patients with DED than healthy people [5, 6]. Excessive inflammation on the eye surface is a critical mechanism of DED [7]. Several proinflammatory cytokines, such as interleukin (IL)-1β, IL-6, IL-8, tumor necrosis factor-α (TNF-α), and matrix metallopeptidase-9 (MMP-9) are often elevated in the tears of patients with DED [8, 9]. Apoptosis plays an important role in driving the progression of DED. Tear hyperosmolarity may cause apoptosis in conjunctival and corneal cells and trigger an inflammatory cascade, further leading to cell death [10, 11]. Therefore, modulation of the apoptotic pathway may inhibit DED progression [12].

Macrophages are important immune cells that act as antigen-presenting cells (APCs) during dry eye injury. Stimulated by hyperosmolarity or proinflammatory cytokines, activated macrophages migrate to draining lymph nodes via afferent lymphatics and induce effector T helper 17 (Th17) cells to migrate to the ocular surface via efferent vessels [13]. Th17 cells further promote epithelial damage by producing proinflammatory cytokines and MMPs, and they also antagonize regulatory T (Treg) cell function, which in turn further releases Th17 and Th1 cells to expand the inflammatory response and cause epithelial damage. M1-type macrophages, which secrete several proinflammatory cytokines and chemokines to promote the inflammatory response, are predominantly found on the ocular surface in DED animal models [14, 15]. Macrophage depletion significantly reduced ocular surface damage, infiltration of other immune cells, and proinflammatory cytokine expression in a DED animal model [16]. Ocular surface changes in DED are associated with increased IL-17 levels in the tears of patients with DED [17]. The neutralization of IL-17 in vivo significantly reduced the severity of DED in mice [18]. Therefore, blocking the activation of macrophages and Th17 cells is a potential strategy for treating DED.

The interaction between intercellular adhesion molecule 1 (ICAM-1) and lymphocyte function-associated antigen 1 (LFA-1) is the key process leading to the vicious inflammatory cycle in DED. When ICAM-1 present on the cell surface of APCs binds to LFA-1 on naive T cells, the interaction promotes the formation of an immunological synapse and initiates T cell differentiation and activation [19, 20]. The latest Food and Drug Administration (FDA)-approved DED drug, Xiidra (lifitegrast), is an LFA-1 antagonist that blocks immunological synapses in patients with DED. However, there are several side effects, including eye irritation, discomfort, changes in taste, and blurred vision [19, 21].

Several animal models have been developed to mimic the pathogenic conditions of DED. Benzalkonium chloride (BAC), a frequently used preservative in eye drops, has been demonstrated to have toxic effects on the eyes [22]. BAC reportedly causes inflammation, instability of the tear film, disruption of the corneal epithelium barrier, and loss of conjunctival goblet cells in several animals, including mice and rats [23, 24]. Models of BAC-induced ocular disruption have been widely used to study inflammatory and evaporative type of DED [25, 26]. Surgical removal of the extra-orbital lacrimal gland produces a model that mimics aqueous tear-deficient dry eye and non-Sjögren’s dry eye type. Based on previous studies, extra-orbital lacrimal gland excision (LGE)-induced DED reduces tear volume by half and causes significant damage to the corneal epithelium [27]. To induce severe, irreversible DED, some researchers injected mice with scopolamine and housed them in a controlled environment chamber (CEC) with room temperature and relatively low humidity [27]. Administration of scopolamine reduces aqueous tear production and the low humidity environment causes excessive evaporation of the tear film. This desiccating stress (DS)-induced animal model is regarded as a mixed evaporative and aqueous tear-deficient dry eye model [27].

As a member of the IL-10 family, IL-20 functions as a proinflammatory cytokine by binding to its complex receptors (IL-20R1/IL-20R2 and IL-20R2/IL-22R1) on target cells. Excessive IL-20 expression is associated with chronic inflammatory diseases, such as rheumatoid arthritis and osteoporosis [28, 29]. We previously found that IL-20 is an upstream factor inducing the expression of several other proinflammatory factors, including IL-1β, IL-6, IL-8, TNF-α, and MMP-9 [28, 30, 31]. Furthermore, IL-20 has been found to promote apoptosis in several cells [32, 33]. Since inflammation and apoptosis are considered to be important mechanisms involved in dry eye, we aimed to explore the role of IL-20 in the pathogenesis of DED and evaluate the therapeutic potential of the anti-IL-20 monoclonal antibody (mAb) 7E for DED treatment.

## Methods

### Study subjects

The study population comprised 40 DED patients and 40 non-DED controls. Tear samples were taken after obtaining written informed consent from all participants between March 2021 and April 2022. The protocol of the clinical study conformed to the ethical guidelines of the 1975 Declaration of Helsinki and was approved by the Ethics Committee of National Cheng Kung University Hospital (IRB No: B-ER-110-038). The diagnosis of DED was based on an ophthalmologist’s assessment of signs and symptoms according to each patient's report of symptoms of ocular irritation as assessed by the Ocular Surface Disease Index (OSDI) score, Schirmer test and tear film breakup time (TBUT). These tests were used to qualify patients for inclusion in the study and for grading DED severity. The exclusion criteria were as follows: age under 20 years old; autoimmune-mediated DED like Sjögren’s syndrome; systemic diseases such as diabetes, rheumatic diseases, blood diseases, and respiratory diseases; history of eye surgery in the previous year; pregnancy; and systemic infections. Participants evaluated as non-DED controls were evaluated with the Ocular Surface Disease Index (OSDI) score, Schirmer’s test and tear film breakup time (TBUT) and demonstrated normal results, including no use of ocular medications. The demographics of the people involved in this study are listed (Additional file 1: Table S1) and details of the analysis of cytokine levels in clinical samples are shown (Additional file 1: Table S2).

### Tear collection

Nonstimulated tear samples were collected from 40 DED patients and 40 non-DED controls by using disposable 20-µl microcapillary tubes (Microcaps 20 μl, Sigma-Aldrich, Cat# P2049). The samples were taken from the marginal tear strip of the lower lid near the lateral canthus without irritating the conjunctiva or cornea according to previous studies [8, 34]. Approximately 10–15 μl of tears were obtained from each participant. The tear samples were placed in microtubes and stored at − 80 °C for further examination. For collecting tears from the animal models, mice were anesthetized and 15 µl of saline were dripped onto the surface of each mouse’s eyes. We used microcapillary tubes to absorb the liquid on the surface of the mouse's eyes from the marginal tear strip of the lower lid near the lateral canthus, and the collected tear samples were immediately stored at − 80 °C. During the tear collection process, we have tried to minimize eye irritation.

### Enzyme-linked immunosorbent assay (ELISA)

Tear samples were diluted five times with 0.1% BSA and analyzed to determine the concentrations of several proinflammatory cytokines, including IL-20 (Sino Biological, Cat# SEK13060), IL-6 (PeproTech, Cat# 900-T16), and IL-8 (PeproTech, Cat# 900-T18), according to the manufacturer’s protocol. Mouse tear was also collected to analyze the levels of IL-20 (R&D Systems, Cat# DY1204), IL-6 (PeproTech, Cat# 900-T50), IL-1β (PeproTech, Cat# 900-T95), and TNF-α (PeproTech, Cat# 900-T54). The protein level of IL-20 (R&D Systems, Cat# DY1102) in the HCE-2 cell lysates and culture medium was also analyzed.

### Benzalkonium chloride (BAC)-induced DED animal model

One hundred wild-type female BALB/c mice (18–20 g, 6–8 weeks old) were obtained from the Laboratory Animal Center (National Cheng Kung University, Tainan, Taiwan) and fed standard laboratory chow and drinking water. The mice were handled according to the guidelines set forth by the Council for International Organization of Medical Sciences on Animal Experimentation (World Health Organization, Geneva, Switzerland) and the guidelines set forth by National Cheng Kung University (IACUC Approval No: 108118). 0.2% BAC (Sigma-Aldrich, #Cat B6295) dissolved in PBS was used to induce DED. In the first week, both eyes of twenty BALB/c mice in the DED groups were topically administered 5 μl of 0.2% BAC twice daily, while the other 5 mice, the healthy control group, were treated with PBS in both eyes. After the induction of DED, the mice were randomly assigned to the various therapeutic groups including the DED + PBS group, DED + mouse IgG (mIgG) group, DED + Xiidra group, and DED + 7E group (anti-IL-20 mAb). 7E and mIgG were dissolved in PBS (pH 7.4) at 800 μg/ml and delivered as a 5 μl drop twice daily. The mice continued to receive BAC administration to the experimental eye throughout the entire period of treatment with therapeutic agents, but the latter was applied approximately 3–4 h after BAC administration to avoid interactions between BAC and the therapeutic agent.

### Extra-orbital lacrimal gland excision (LGE)-induced DED animal model

To establish aqueous tear-deficient DED, sixty-five female C57BL/6 mice were used with an average body weight of 22–25 g (8 weeks old). The mice were handled according to the guidelines set forth by the Council for International Organization of Medical Sciences on Animal Experimentation and the guidelines set forth by National Cheng Kung University (IACUC Approval No: 110345). Surgery was performed on day 1, the mice were IP injected with Zoletil (50 mg/kg) and Rompun (5.8 mg/kg) to anesthetize and the extra-orbital lacrimal gland was surgically removed (Additional file 1: Fig. S1), and the wound was sutured. For the sham group, the incisions were made and the extra-orbital lacrimal glands were partially exposed and not removed. The operation was carried out in a near-sterile state. After the operation, the mice were kept warm, and the mice will not be put back into the cage until the mice wake up. Drug treatment was carried out on day 8, and mice were administered topically twice daily until day 14.

### Desiccating stress (DS)-induced DED animal model

Fifty-six female C57BL/6 mice (22–25 g; 8 weeks old) were placed in a controlled environment chamber (CEC) (relative humidity < 25%, 24–25 °C temperature, 24 h per day) to induce desiccating stress so that the mice’s tears were excessively evaporated as previously reported [35]. The experimental procedures were according to the guidelines set forth by the Council for International Organization of Medical Sciences on Animal Experimentation and the guidelines set forth by National Cheng Kung University (IACUC Approval No: 110345). During the experiment, the animals’ behavior, food, and water intake were not restricted. Mice were exposed to CEC for 14 days and scopolamine hydrobromide (0.375 mg/150 µl) was subcutaneously injected into mice twice daily on days 2 and 4. The drug treatment was started on day 8 of the experiment, and the drug was directly applied topically to the eyes of the mice, three times a day. Uninduced mice were served as controls.

### Tear production measurement

Tear volume was measured by SMTube test at the same time of day in the standard environment as previously described [36]. Briefly, an SMTM was immersed in the inferior tear meniscus of the eye by gently touching the eyelid and ocular surface. The wetted length (millimeter) of the tube was read after 30 s.

### Corneal fluorescein staining

Each eye was stained with fluorescein to visualize corneal epithelial damage at the same time of day. In brief, 1 μl of 0.1% liquid fluorescein (Sigma-Aldrich, Cat# F6377) was dropped into the conjunctival sac of mice. After ninety seconds, corneal epithelial damage was visualized with a cobalt blue filter under a Micron IV (Phoenix MICRON, Phoenix Technology Group). Each eye image was evaluated, and the fluorescein score was quantified blindly by four individuals. The fluorescein score was analyzed as follows: 0, no green staining; 0.5, slightly green dotted staining; 1, diffuse dot-like green staining; 2, green stained area less than one-third of the corneas; 3, green stained area more than one-third of the corneas; and 4, green stained area more than two-thirds of the corneas.

### Cell culture

An immortalized human corneal epithelial cell line (HCE-2) (ATCC® CRL-11135™) was originally purchased from American Type Culture Collection. Cells were cultured in keratinocyte-serum-free medium (Thermo Fisher Scientific, Cat# 17005042) supplemented with additives including 0.05 mg/ml bovine pituitary extract, 5 ng/ml epidermal growth factor, 500 ng/ml hydrocortisone (Sigma-Aldrich, Cat# H0888), 10% FBS, 1× antibiotic–antimycotic (GeneDireX, Cat# CC501-0100), and 0.005 mg/ml insulin (Santa Cruz Biotechnology, Cat# sc-360248). Bone marrow-derived macrophages (BMDMs) were isolated and cultured in DMEM/HG (GeneDireX, Cat# CC126-1010) supplemented with10% FBS, and 1× antibiotic–antimycotic.

### Immunohistochemistry

Eyes tissues were fixed in 4% paraformaldehyde solution and were proceed with dehydration and embedding according to common methods by Human Biobank in National Cheng Kung University Hospital. Paraffin-embedded tissue sections (4 μm) were deparaffinized and rehydrated and subjected to heat-induced antigen retrieval by citrate buffer (pH 6.4) for 95 °C 30 min. Nonspecific binding was blocked by treatment with blocking reagent (Thermo Fisher Scientific, Cat# 003118). The sections were incubated with anti-IL-20 antibody (7E) or anti-MUC5AC antibody (Abcam, Cat# ab3649) at 4 °C overnight. 7E were prepared as previously described [37, 38]. The next day, the sections were washed with PBS and incubated with the secondary antibody for 1 h. The reaction was detected using AEC chromogen stain (ScyTek Laboratories, Cat# ACG500), and the nuclei were counterstained with hematoxylin (ScyTek Laboratories, Cat# HMM500). Pictures were taken under an inverted fluorescence microscope IX71 (Olympus).

### Immunocytochemistry (ICC)

Cells were incubated with anti-BAX antibody (Biolegend, Cat# 633602) or anti-BCL-2 antibody (Biolegend, Cat# 633502) or anti-phospho-NFAT5 (Ser145) antibody (Invitrogen, Cat# PA5-105436) or anti-IL-20 antibody (7E) or anti-IL-20R1 antibody (Abcam, Cat# ab90935) or anti-IL-20R2 antibody (Abcam, Cat# ab95824) or anti-IL-22R1 antibody (Abcam, Cat# ab5984) at 4 °C overnight. The next day, the sections were washed with PBS and incubated with the secondary antibody for 1 h. Reactions were detected using AEC chromogen stain (ScyTek Laboratories, Cat# ACG500) and nuclei were counterstained with hematoxylin (ScyTek Laboratories, Cat# HMM500).

### Immunofluorescence (IF)

Samples were incubated with anti-F4/80 antibody (Cell Signaling Technology, Cat# 70076) or anti-phospho-NFAT5 (Ser145) antibody (Invitrogen, Cat# PA5-105436) and anti-IL-20 antibody (7E) at 4 °C overnight. Next day, the sections were incubated for 1 h with Alexa Fluor®488-conjugated anti-mouse secondary antibody (Jackson ImmunoResearch Labs, Cat# 115-545-003) and Alexa Fluor®594-conjugated anti-rabbit secondary antibody (Jackson ImmunoResearch Labs, Cat# 111-585-003), and finally mounted with DAPI (Vector Laboratories, Cat# H-1200). Pictures were taken under an inverted fluorescence microscope IX71 (Olympus).

### Cell viability

Cell viability was measured through Cell Counting Kit-8 (Abbkine, Cat# KTC011001) according to the manufacturer’s protocol. HCE-2 cells were treated with 10 μl per 100 μl media of CCK-8 reagent and were incubated at 37 °C for 2–3 h and were measured absorbance at 450 nm.

### Flow cytometry

Murine bone marrow-derived M0-type macrophages were treated with untreated (control), LPS + IFN-γ (M1), 80 mM NaCl + PBS, 80 mM NaCl + Xiidra, 80 mM NaCl + mIgG, 80 mM NaCl + 7E for 24 h and were collected and stained with FITC anti-F4/80 (Biolegend, Cat# 123108), APC anti-CD86 (Biolegend, Cat# 105012), and PE anti-CD206 (Biolegend, Cat# 141706) antibodies. The expression of F4/80, CD86, and CD206 was analyzed by a CytoFLEX (Beckman Coulter) flow cytometer. Annexin V-FITC Apoptosis Staining/Detection Kit (Abcam, Cat# ab14085) was applied to detect cell death according to the manufacture’s protocol by CytoFLEX (Beckman Coulter) flow cytometer. Cells isolated from conjunctiva or draining lymph nodes were incubated in the RPMI1640 culture medium with PMA (50 ng/ml), ionomycin (1 µg/ml), and Brefeldin A (Golgi Plug) (10 µg/ml). Five hours later, cells were harvested and stained with PE/Cyanine7 anti-mouse CD4 Antibody (Biolegend, Cat# 100422) and fixed by 4% paraformaldehyde. Cells were then treated with permeabilization by BD Cytofix/Cytoperm Plus (BD, Cat# 555028) and stained with PE anti-mouse IFN-γ antibody (Biolegend, Cat# 505807) and PerCP anti-mouse IL-17A antibody (Biolegend, Cat# 506943) and analyzed by CytoFLEX flow cytometer.

### H&E staining and periodic acid–schiff (PAS) staining

Tissue sections were deparaffinized, rehydrated, and stained with PAS staining kit (Sigma-Aldrich, Cat# 1016460001) or hematoxylin and eosin stain according to common methods by Human Biobank in National Cheng Kung University Hospital.

### Hyperosmotic stress-induced cell model

Hyperosmolar conditions were achieved by the addition of different amounts of sodium chloride (NaCl) into the culture medium according to a protocol described in previous studies [39, 40]. A osmometer (LÖSER) was used to measure approximately 350 mOsm for 40 mM NaCl and approximately 400 mOsm for 80 mM NaCl.

### TUNEL assay

Paraffin sections from mice were deparaffinized and rehydrated. The apoptotic cells were detected by staining with DeadEnd™ Fluorometric TUNEL System (Promega, Cat# G3250) according to the manufacture’s protocol.

### Real-time PCR

Total RNA was extracted from frozen eye samples or cells using Trizol reagent (Sigma-Aldrich, Cat# T9424) and reverse transcription was performed with reverse transcriptase (Invitrogen, Cat# 28025013) according to the manufacturer’s protocol. The expression levels of *Il20*, *Il20r1*, *Il20r2*, *Il22ra*, *F4/80*, *Nos2*, *Arg1*, *Icam1*, *Il6*, *Il8*, *Bax*, *Bcl2*, *Il1b*, *Tnfa*, *Mcp1*, *Il10*, *Mmp9*, *Cd4*, *Il17a*, and *Ifnγ* were amplified on a StepOnePlus (Applied Biosystems), with SYBR Green (Thermo Fisher Scientific, Cat# 4385610) for quantitative analysis normalized with glyceraldehyde phosphate dehydrogenase (*Gapdh*), as an internal control. Relative multiples of change in mRNA expression were determined by calculating 2^−ΔΔCt^. The sequence of each primer used in this study are listed (Additional file 1: Table S3).

### Western blot

Thirty micrograms of protein were loaded into SDS-PAGE gel (10 or 15%) and ran for 3 h. Protein gel was transferred to a 0·45 µm PVDF membrane and was blocked by 5% milk or 5% BSA dissolved in TBST. Antibodies used in this study include anti-NFAT5 antibody (Invitrogen, Cat# PA1-023), anti-phospho-NFAT5 (Ser145) antibody (Invitrogen, Cat# PA5-105436), anti-IL-20 antibody (7E), and anti-GAPDH antibody (Proteintech, Cat#, 10494-1-AP). The original western blot images are provided (Additional file 2).

### Statistical analysis

Statistical analysis was performed using GraphPad Prism version 6 (GraphPad Software, USA). Experimental data are presented as the mean ± SEM. GraphPad Prism software was used to process initial data and graphs. Comparisons between two groups were analyzed by an unpaired two-sided Student’s t-test. One-way ANOVA was used to compare data among groups in experiments including three or more groups. *p* < 0.05 was considered significant (*p < 0.05, **p < 0.01, ***p < 0.001, and ****p < 0.0001).

## Results

### IL-20 is upregulated in the tears of patients with DED and the corneas of DED mouse models

**Fig. 1:**
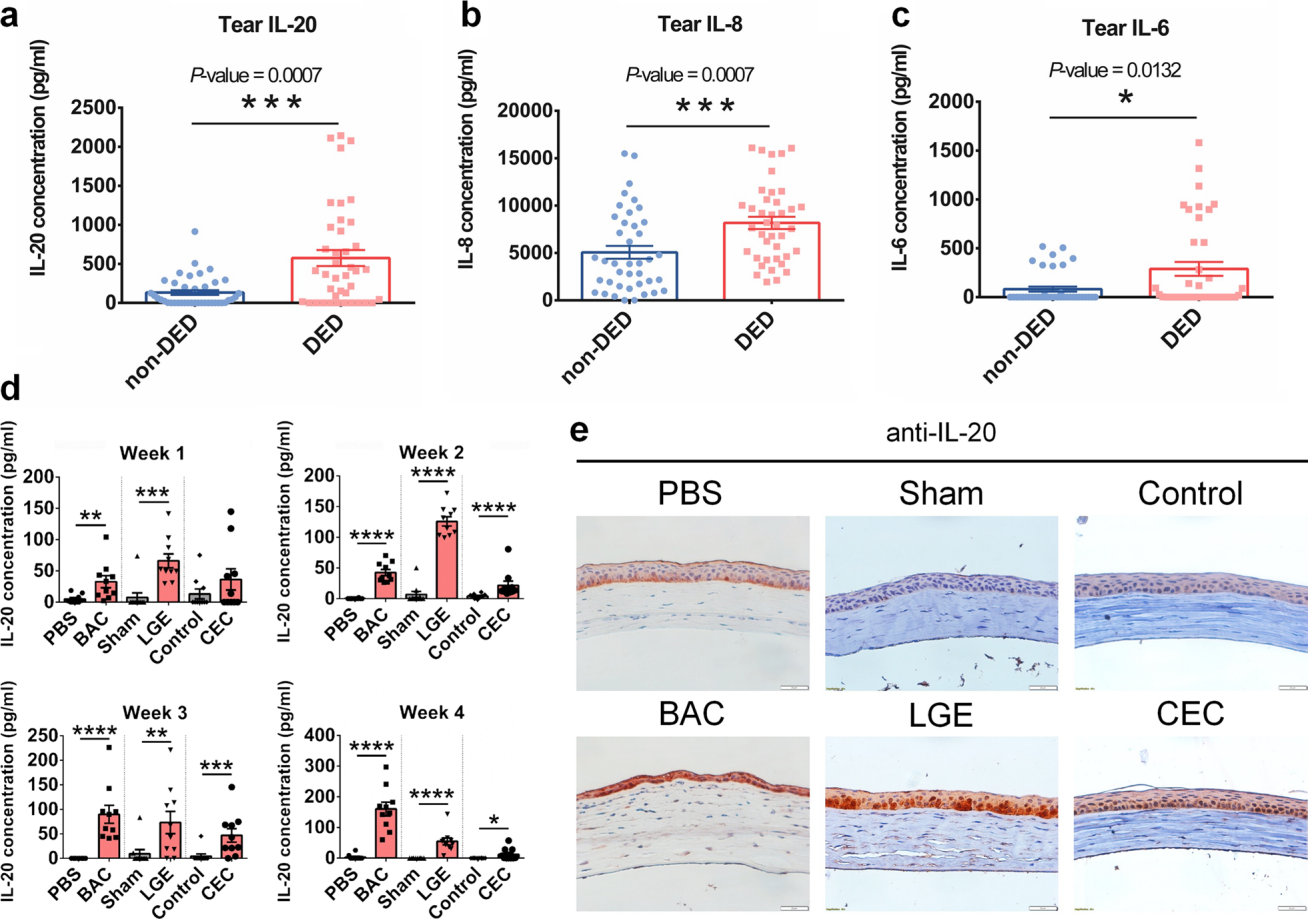
Elevated IL-20 levels in the tears of patients with DED and DED animal model corneas. **a**–**c** Tear samples were harvested from non-DED controls (n = 33) and patients with DED (n = 40). Tear IL-20, IL-6, and IL-8 levels were measured using ELISAs. Mann–Whitney test, *p < 0.05 and *** *p* < 0.001. Data are shown as the mean ± SEM. **d** Mice were topically administered PBS or BAC twice daily for 4 weeks (for each group, n = 10). Bilateral extra-orbital lacrimal gland excision was applied to induce an aqueous tear-deficient DED animal model for 4 weeks, sham was used for control mice (for each group, n = 10). Mice were housed in a CEC with low humidity for 28 days for DS-induced DED; non-induced mice served as healthy controls (for each group, n = 10). Mice were harvested tear for each week and analyzed for protein level of IL-20 by ELISA. One-way ANOVA, **p < 0.01, ***p < 0.001, and ****p < 0.0001. Data are shown as the mean ± SEM. **e** Immunohistochemical (IHC) staining was used to detect the protein expression of IL-20 in the mouse cornea. The reaction was detected using staining with the chromogen AEC (red), and the nuclei were counterstained with hematoxylin (blue). Original magnification: ×400. The experiments in **e** were independently repeated three times, with similar results, and the data of one representative experiment is shown. *DED* dry eye disease, *ELISA* enzyme-linked immunosorbent assay, *PBS* phosphate-buffered saline, *BAC* benzalkonium chloride, *CEC* controlled environment chamber, *LGE* lacrimal gland excision, *DS* desiccating stress

To investigate the involvement of IL-20 in the pathogenesis of DED, we examined IL-20 expression in tear samples from patients with DED (n = 40) and non-DED controls (n = 40). The IL-20 tear levels were higher in patients with DED than in non-DED controls, indicating that IL-20 may be involved in the pathogenesis of this disease (Fig. 1a). In addition, the tear levels of IL-8 and IL-6 were also higher in the DED groups than in the non-DED control group (Fig. 1b, c). To investigate whether IL-20 was involved in the pathogenesis of this condition, we established three DED animal models to mimic human DED. Firstly, 0.2% BAC was topically administered to mice twice daily for 2 weeks. BAC has been widely used to model human evaporative DED by triggering inflammation, immune cell infiltration, tear film instability, and conjunctival goblet cell loss [25, 41]. The main clinical characteristics of DED, including decreased tear volume and corneal epithelium damage, were observed in the BAC model (Additional file 1: Fig. S2a–c). Secondly, a severe aqueous tear-deficient DED animal model was established via extra-orbital LGE [42]. Thirdly, mice were injected with scopolamine and housed in a controlled environment chamber (CEC) with low humidity to induce desiccating stress (DS) to mimic human mixed evaporative and aqueous tear-deficient DED. The corneal *IL20* transcript levels were significantly upregulated (Additional file 1: Fig. S2d). Similar to the results of the analysis in humans, IL-20 was significantly upregulated in tears from these DED animal models (Fig. 1d). However, other proinflammatory cytokines including IL-1β, IL-6, and TNF-α were only significantly upregulated in the LGE model (Additional file 1: Figs. S3, S4). IL-20 were also highly increased in the corneas of DED mice compared to those of healthy control mice on day 14 (Fig. 1e).

### IL-20 is induced under hyperosmotic stress in corneal epithelial cells

**Fig. 2:**
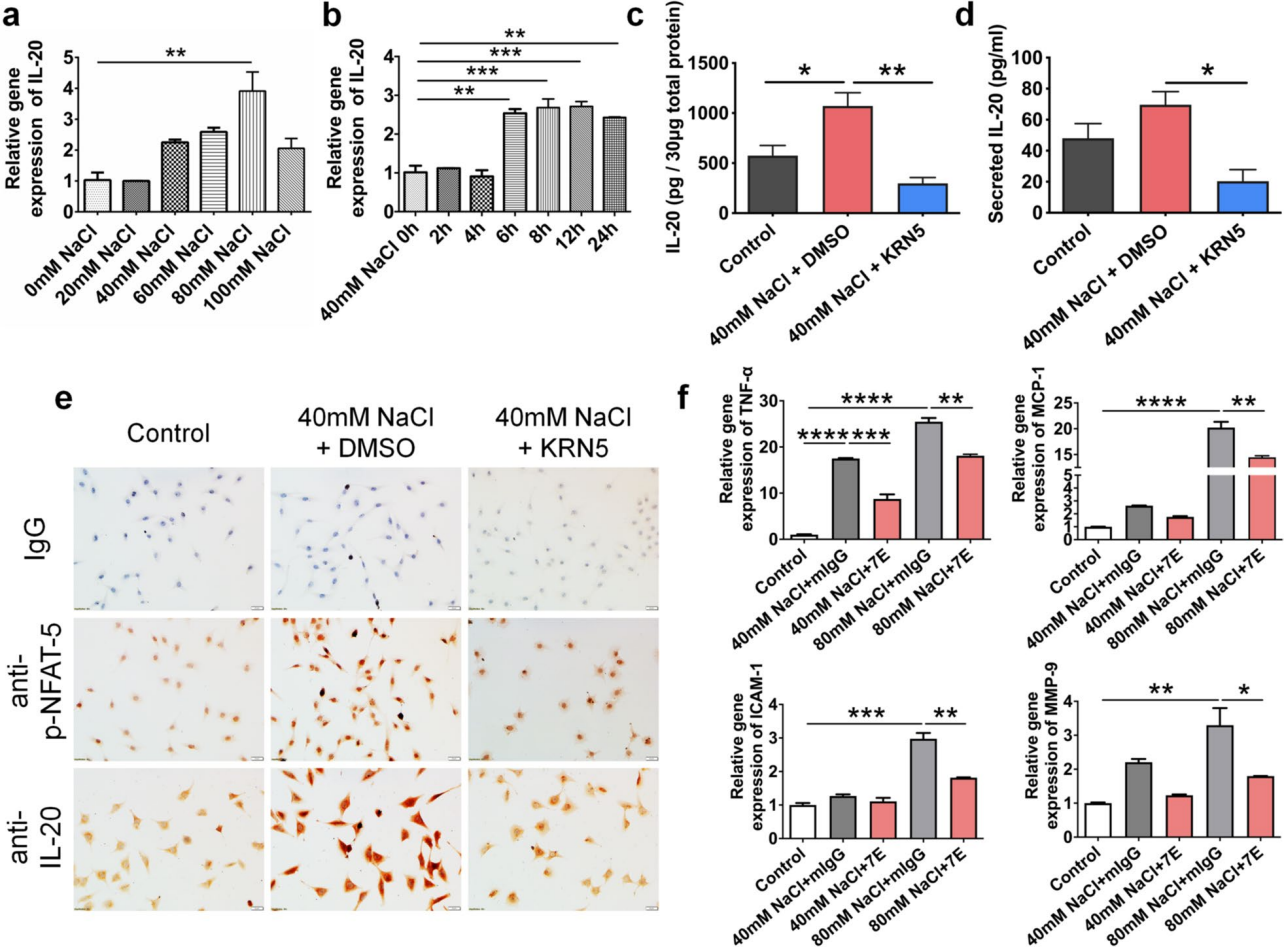
IL-20 expression was upregulated by NFAT5 activation under hyperosmotic stress. The hyperosmotic condition was achieved by adding various amounts of sodium chloride (NaCl) into the culture medium of HCE-2 cells. **a**, **b** Cells were treated with 0 mM, 20 mM, 40 mM, 60 mM, 80 mM, and 100 mM NaCl for 8 h. 40 mM NaCl was added into the media for 0, 2, 4, 6, 8, 12, and 24 h. The mRNA transcript of *IL-20* was analyzed using real-time PCR with specific primers. *GAPDH* was used as an internal control. One-way ANOVA, **p < 0.01 and ***p < 0.001. Data are shown as the mean ± SEM. **c**, **d** HCE-2 cells were cultured without NaCl or with 40 mM NaCl + DMSO or 40 mM NaCl + KRN5 (an inhibitor of NFAT5) for 24 h. Total cell lysates and culture medium were harvested, and the IL-20 protein level was determined using an ELISA. One-way ANOVA, *p < 0.05 and **p < 0.01. Data are shown as the mean ± SEM. **e** Immunocytochemistry was also applied to analyze the protein levels of IL-20 and phospho-NFAT5 (Ser145). The reaction was detected using the chromogen AEC (red), and the nuclei were counterstained with hematoxylin (blue). Original magnification: ×200. **f** HCE-2 cells received control or hyperosmotic stress treatments (40 mM or 80 mM NaCl) to mimic dry eye conditions. mIgG (4 µg/ml) or 7E (4 µg/ml) were added to the medium for 8 h. The mRNA transcripts of *TNF-α*, *MCP-1*, *ICAM-1*, and *MMP-9* were analyzed using real-time PCR with specific primers. *GAPDH* was used as an internal control. One-way ANOVA, *p < 0.05, **p < 0.01, ***p < 0.001, and ****p < 0.0001. Data are shown as the mean ± SEM. The experiments in **a**–**f** were independently repeated three times with similar results, and the data of one representative experiment is shown. *NFAT5* nuclear factor of activated T‑cells 5, *ELISA* enzyme-linked immunosorbent assay

Previous studies indicated that tear hyperosmolarity is the key mechanism contributing to ocular surface inflammation and damage in DED [10, 43]. Corneal epithelial cells are significantly affected when tear osmolarity is high. Results from the DED animal models also showed increases in both tear osmolarity and IL-20 (Additional file 1: Fig. S4). To test whether IL-20 is altered by hyperosmolarity, we incubated HCE-2 cells, a human corneal epithelial cell line, with sodium chloride to induce hyperosmotic stress and mimic dry eye conditions in vitro. The transcript and protein levels of IL-20 were significantly upregulated under hyperosmotic stress conditions (Fig. 2a, b, Additional file 1: Fig. S5a). The nuclear factor of activated T‑cells 5 (NFAT5) is an osmotic sensitive transcription factor mediating the expression of genes involved in cell survival under hypertonic conditions [44]. KRN5, an inhibitor of NFAT5, abolished hyperosmolarity-induced IL-20, suggesting that NFAT5 mediates IL-20 upregulation under hyperosmotic stress conditions (Fig. 2c–e, Additional file 1: Figs. S5b, S6). Additionally, hyperosmotic stress-induced TNF-α, MCP-1, ICAM-1, and MMP-9 expression was significantly downregulated by the anti-IL-20 mAb 7E in HCE-2 cells in the 7E-treated group compared with the mIgG-treated group (Fig. 2f). We then analyzed the direct effects of IL-20 on the regulation of several key proinflammatory cytokines in HCE-2 cells. The proteins and transcripts of IL-20, IL-20R1, IL-20R2, and IL-22R1 were expressed in HCE-2 cells (Additional file 1: Fig. S7), suggesting that corneal cells could be targeted by IL-20 in an autocrine manner. In response to IL-20, gene expression of *IL-1β*, *IL-6*, *IL-8*, *ICAM-1*, and *MMP-9* was induced in HCE-2 cells (Additional file 1: Fig. S8a). BAC (0.001%) significantly induced important proinflammatory cytokines, including IL-1β, IL-6, IL-8, and ICAM-1 in HCE-2 cells, and these effects could be reversed by 7E treatment (Additional file 1: Fig. S8b).

### 7E treatment suppresses hyperosmotic stress-induced activation of macrophages

**Fig. 3:**
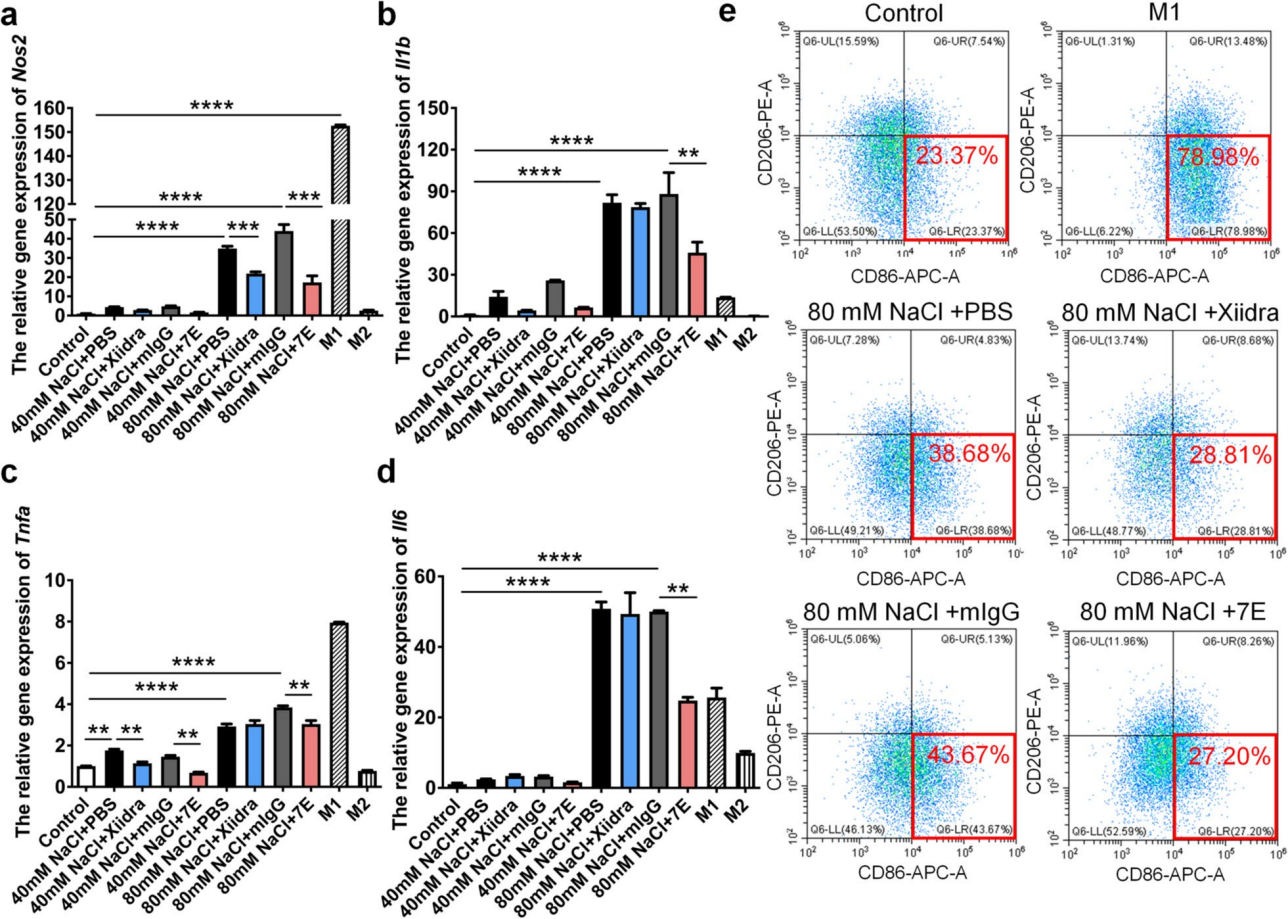
7E protected macrophages from hyperosmotic stress-induced activation. **a**–**d** Murine bone marrow cells were isolated for cell culture and treated with M-CSF (100 ng/ml) for 5 days to differentiate into BMDMs. Cells were further treated with 40 mM or 80 mM NaCl to mimic dry eye conditions. Drugs including Xiidra and 7E were added into the medium for 24 h, PBS and mIgG were used as control groups. Cells were harvested and analyzed for the expression levels of several proinflammatory factors including *Nos2*, *Il1b*, *Tnfa*, and *Il6*. *Gapdh* was used as an internal control. One-way ANOVA, **p < 0.01, ***p < 0.001 and ****p < 0.0001. Data are shown as the mean ± SEM. **e** Murine bone marrow-derived M0-type macrophages were treated LPS + IFN-γ (M1), 80 mM NaCl + PBS, 80 mM NaCl + Xiidra, 80 mM NaCl + mIgG, 80 mM NaCl + 7E for 24 h and analyzed for expression of F4/80, CD86, and CD206 by flow-cytometry. The experiments in **a**–**d** were repeated three times independently with similar results, and the data of one representative experiment was shown. *M-CSF* macrophage colony-stimulating factor, *BMDMs* bone marrow-derived macrophages, *LPS* lipopolysaccharide, *IFN-γ* interferon-γ

Macrophages are key immune cells recruited to the cornea during ocular surface disease. Under pathological conditions, macrophages are stimulated by hyperosmotic stress, initiate inflammatory responses [45], and are polarized toward the M1 phenotype [35, 46]. To test whether IL-20 alters macrophage activation, we incubated murine BMDMs with hyperosmotic stress and analyzed the effects of 7E. Results from qRT-PCR showed that hyperosmotic stress upregulated transcript levels of *Nos2* (M1-type macrophage marker), *Il1b*, *Tnfa*, and *Il6* (Fig. 3a–d). However, 7E treatment significantly suppressed hyperosmotic stress-induced upregulation of these proinflammatory factors (Fig. 3a–d) and the polarization of macrophages toward M1-type (Fig. 3e).

### 7E protects corneal epithelial cells from hyperosmotic stress-induced cell death by regulating BAX and BCL-2 in vitro

**Fig. 4:**
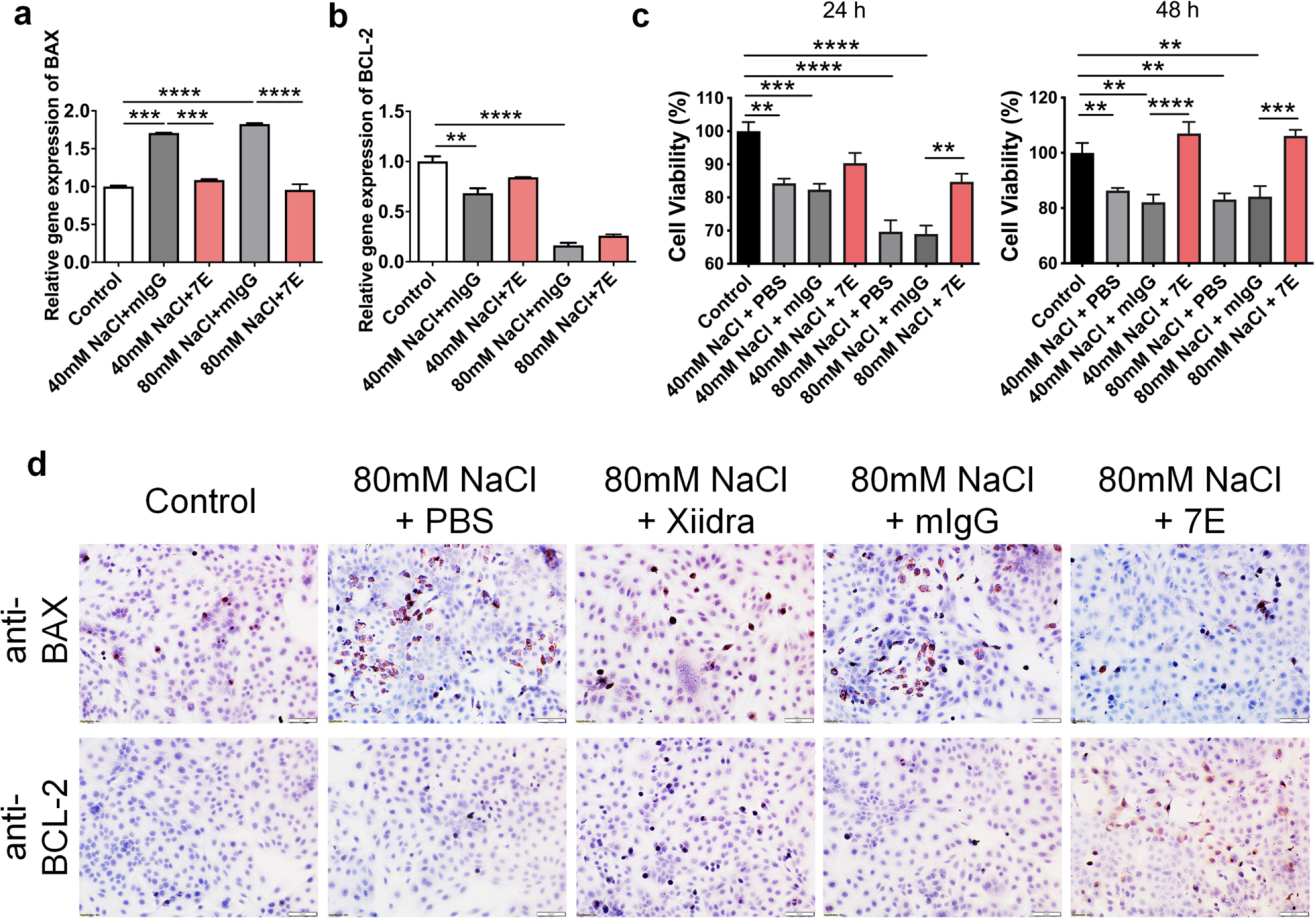
7E protected corneal epithelial cells from hyperosmotic stress-induced cell death. HCE-2 cells were subjected to control, 40 mM + mIgG (4 µg/ml), 40 mM + 7E (4 µg/ml), 80 mM + mIgG, and 80 mM + 7E for 8 h. **a**, **b** The mRNA transcripts of BAX and BCL-2 were analyzed by real-time PCR with specific primers. GAPDH was used as an internal control. One-way ANOVA, **p < 0.01, ***p < 0.001, and ***p < 0.0001. Data are shown as the mean ± SEM. **c** CCK-8 assay was applied to analyze the cell viability of HCE-2 cells treated with different groups for 24 h or 48 h. One-way ANOVA, **p < 0.01, ***p < 0.001, and ****p < 0.0001. Data are shown as the mean ± SEM. **d** Immunocytochemistry staining of BAX and BCL-2 of different groups was analyzed. The reaction was detected by the chromogen AEC (red), and the nuclei were counterstained with hematoxylin (blue). Original magnification: ×200. The experiments in **a**–**d** were repeated three times independently with similar results, and the data of one representative experiment was shown. *CCK-8* cell counting kit-8

Cell death occurs in chronic hyperosmotic conditions and may cause loss of cornea epithelium integrity, which is a hallmark of DED. Hyperosmotic stress upregulated BAX and downregulated BCL-2, which was reversed during 7E treatment (Fig. 4a, b). To investigate whether 7E protects HCE-2 cells from hyperosmotic stress- or BAC-induced cell death, we performed a cell viability assay and found that the 7E-treated group showed higher cell viability than the mIgG-treated control group (Fig. 4c, Additional file 1: Fig. S9a). Immunostaining showed that 7E downregulated the protein level of BAX and upregulated the protein expression of BCL-2 to protect cells from hyperosmotic stress-induced cell death (Fig. 4d). To further clarify the direct effects of IL-20 on HCE-2 cells, we performed a PI/Annexin V assay to verify whether IL-20 directly induces cell death in corneal epithelial cells. FACS analysis showed that IL-20 treatment decreased the number of healthy living cells and increased the numbers of apoptotic and necrotic cells (Additional file 1: Fig. S9b, c).

### 7E mitigates disease severity in the BAC-induced inflammatory DED model

**Fig. 5:**
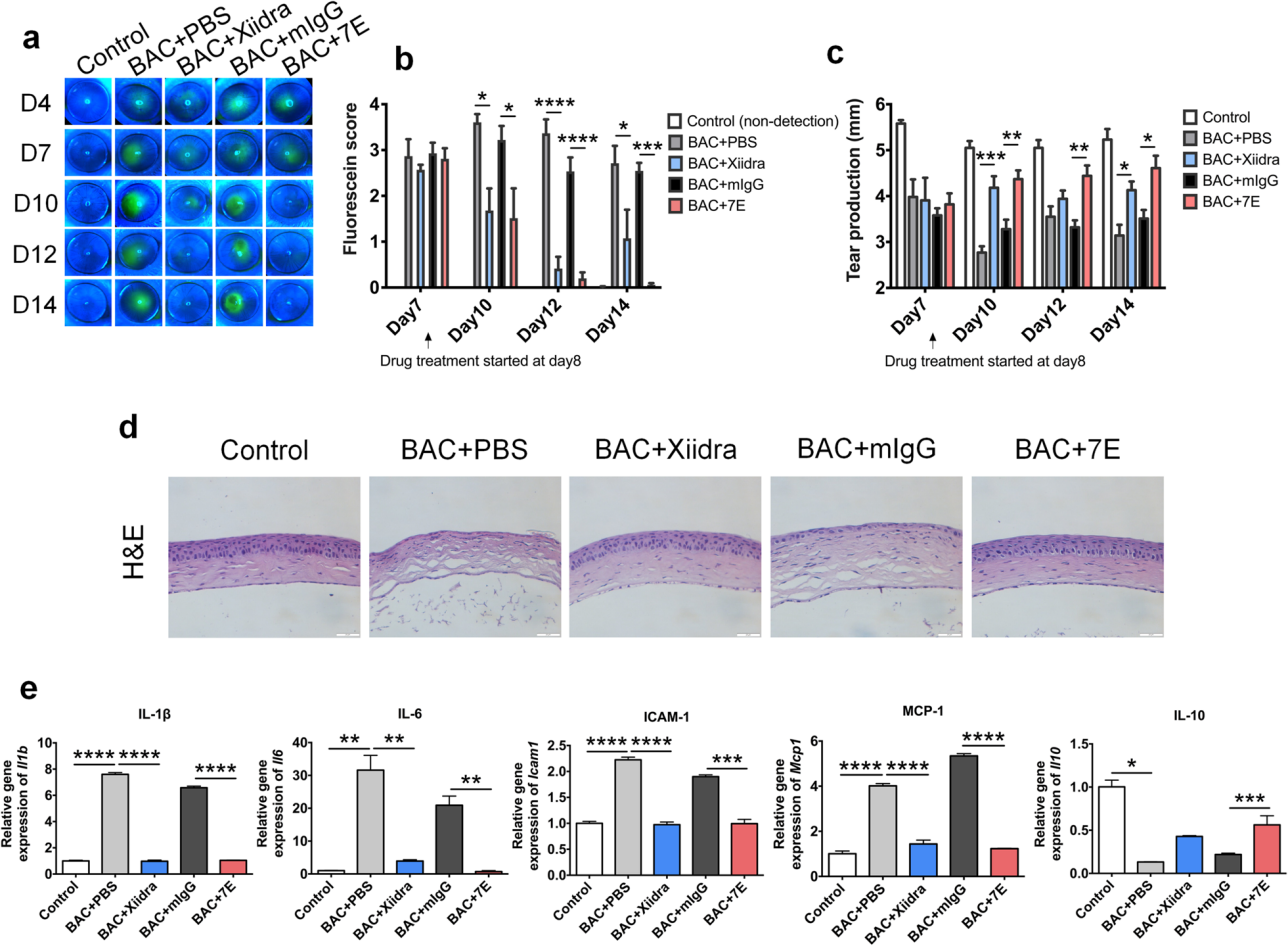
7E treatment improved BAC-induced dry eye symptoms in mice. Mice were topically administered PBS or BAC twice daily for 2 weeks to induce DED. PBS, Xiidra, mIgG, or 7E was administered twice daily beginning on day 8 (n = 5, each group). **a** Corneal fluorescein staining was performed to analyze the integrity of the corneal epithelium. Representative images were taken with a cobalt blue filter by a Micron IV. **b** The fluorescein scores were blindly evaluated by four individuals. One-way ANOVA, *p < 0.05, ***p < 0.001, and ****p < 0.0001. Data are shown as the mean ± SEM. Arrow indicates the treatment started on day 8. **c** Tear production was measured at the same time of day in the standard environment. One-way ANOVA, *p < 0.05, **p < 0.01, and ***p < 0.001. Data are shown as the mean ± SEM. **d** H&E staining was performed to observe corneal morphology. Original magnification: ×400. **e** The corneal mRNA transcripts of *Il1b*, *Il6*, *Icam1*, *Mcp1*, and *Il10* were analyzed by real-time PCR with specific primers. *Gapdh* was used as an internal control. One-way ANOVA, *p < 0.05, **p < 0.01, ***p < 0.001, and ****p < 0.0001. Data are shown as the mean ± SEM. The experiments in **a**–**e** were repeated three times independently with similar results, and the data of one representative experiment was shown. *BAC* benzalkonium chloride, *DED* dry eye disease

Based on our observation that IL-20 was upregulated in DED mice, we investigated the involvement of IL-20 in DED progression and examined the therapeutic effects of 7E on the BAC-induced evaporative dry eye mouse model. We topically administered 0.2% BAC to mice twice daily for 14 days. Drugs, including PBS, Xiidra (lifitegrast 5%), mIgG, and 7E, were topically applied twice daily starting on day 8. The eyes of each mouse were stained with fluorescein to visualize corneal epithelial damage on days 4, 7, 10, 12, and 14 at the same time of day, and the staining was quantified to determine the fluorescein score (Fig. 5a, b). Both the Xiidra- and 7E-treated groups showed reduced damage to the corneal epithelium on days 10, 12, and 14 compared with the PBS- and mIgG-treated control groups. Tear production tests were performed on days 7, 10, 12, and 14 for each group (Fig. 5c). The 7E group showed an increase in tear production on days 10, 12, and 14 compared to the mIgG-treated control group. The Xiidra group increased tear production on days 10 and 14 compared to the PBS-treated control group. Interestingly, topical application of 7E to the eye also reduced systemic levels of IL-20 and IL-6 (Additional file 1: Fig. S10a, b). The BAC + Xiidra and BAC + 7E groups showed a decreased *Bax/Bcl2* ratio in the cornea compared to the BAC + PBS and BAC + mIgG control groups (Additional file 1: Fig. S10c). H&E staining of the mouse cornea showed that both 7E and Xiidra treatment reduced the inflammatory response (Fig. 5d), which is characterized by decreasing layers of the corneal epithelium and infiltrating immune cells. The main mucin protein in the eye, MUC5AC, was also maintained in the BAC + 7E group (Additional file 1: Fig. S11). These results suggested that 7E effectively maintained the mucin content in conjunctival goblet cells. Thus, we further investigated whether Xiidra and 7E treatments maintain the mucin content by inhibiting BAC-induced cell apoptosis. Numerous TUNEL-positive (apoptotic) cells were observed in the cornea and goblet cell-rich conjunctival tissue in the BAC + PBS and BAC + mIgG groups. Apoptotic cell numbers appeared reduced in the BAC + Xiidra and BAC + 7E groups at the conjunctival and corneal sites (Additional file 1: Fig. S10d, e), demonstrating that 7E effectively inhibited cell apoptosis to maintain mucin content.

### 7E reduces macrophage infiltration and inhibits corneal inflammation

Macrophage infiltration has been implicated in immune-mediated dry eye injury [47]. We noticed the co-localization of F4/80 and IL-20 in the corneal stroma in BAC-induced DED cornea (Additional file 1: Fig. S12a). To verify whether infiltrated macrophage numbers decreased after 7E treatment in BAC-induced DED mice, we performed immunofluorescence staining for the macrophage marker F4/80 in the corneas of BAC-induced DED mice. The number of infiltrated macrophages was significantly reduced in the BAC + 7E group compared with the BAC + mIgG group (Additional file 1: Fig. S12b, c). Real-time PCR was performed to analyze the gene expression of F4/80 and markers of M1- and M2-type macrophages in corneal tissue. The dry eye condition significantly induced the M1-type macrophage marker, inducible nitric oxide synthase (*iNOS/Nos2*), rather than the M2-type macrophage marker, arginase 1 (*Arg1*) (Additional file 1: Fig. S12d). Since M1-type macrophages often cause severe inflammatory responses, we analyzed several proinflammatory mediators in BAC-induced DED mice. The gene expression levels of proinflammatory cytokines *Il1b*, *Il6*, *Icam1*, and *Mcp1* were significantly upregulated, while that of the anti-inflammatory cytokine *Il10* was downregulated in the BAC + PBS and BAC + mIgG groups (Fig. 5e). However, the gene expression of proinflammatory cytokines was suppressed in the BAC + 7E group compared with the BAC + mIgG control group, while that of *Il10* was elevated (Fig. 5e).

### 7E reduces Th17 cell population in the LGE-induced aqueous tear-deficient DED model

**Fig. 6:**
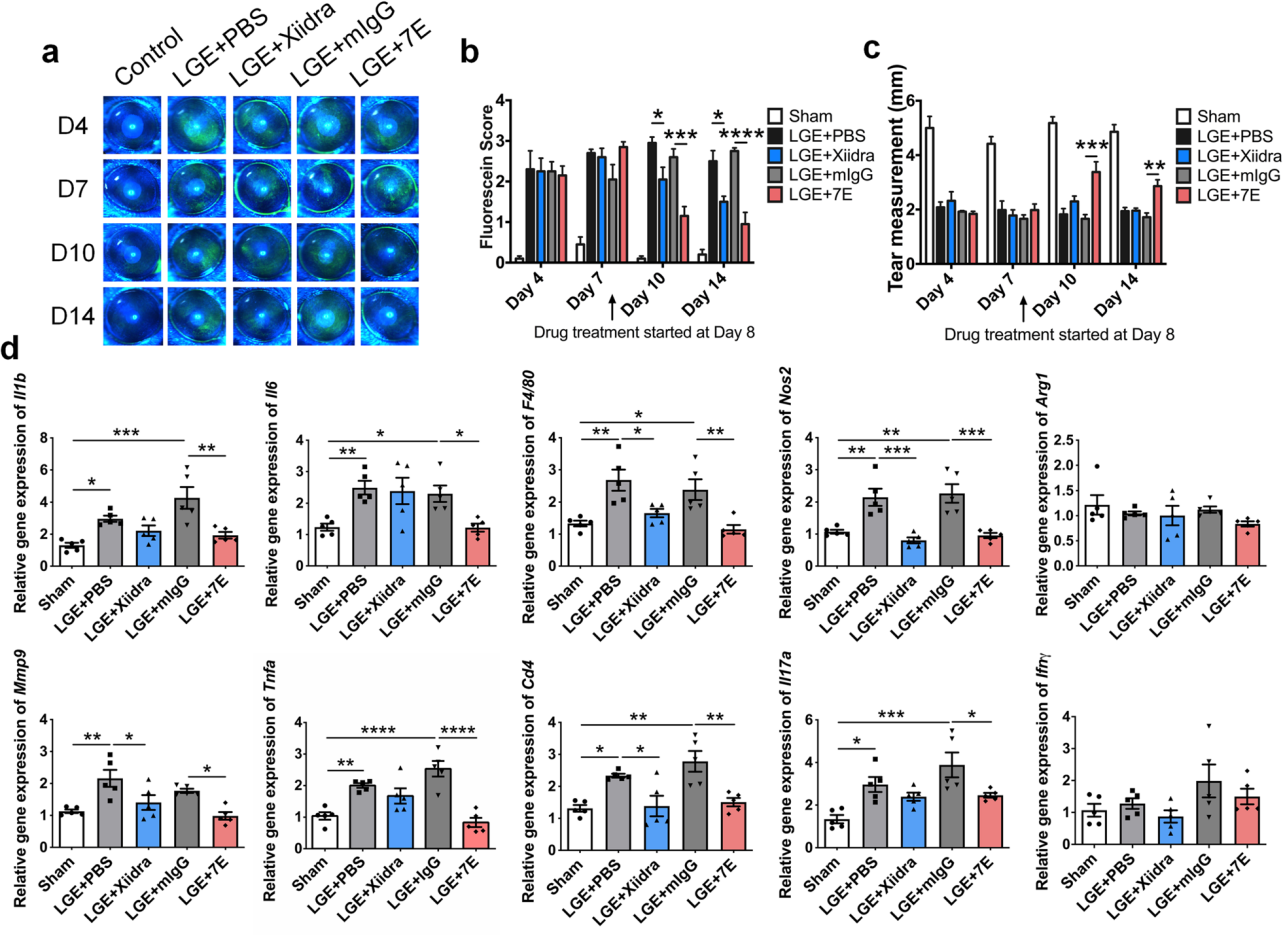
7E treatment produced protective effects against LGE-induced aqueous tear-deficient DED. A mouse model of aqueous tear-deficient DED induced by surgical removal of the extra-orbital lacrimal glands on the first day of the experiment. Sham surgery was used as control mice. Mice were treated with drugs, including PBS, Xiidra, mIgG, 7E (each group, n = 5) twice a day starting on day 8, and all mice were sacrificed on day 14. **a** Corneal epithelial integrity was analyzed by corneal fluorescein staining on days 4, 7, 10, and 14. Representative images were taken by Micron IV with a cobalt blue filter. **b** Fluorescein scores were blindly assessed by four individuals. One-way ANOVA, *p < 0.05, ***p < 0.001, and ****p < 0.0001. Data are shown as the mean ± SEM. **c** Tear production was measured at the same time on days 4, 7, 10, and 14. One-way ANOVA, **p < 0.01 and ***p < 0.001. Data are shown as the mean ± SEM. **d** Corneal mRNA transcripts of *Il1b*, *Il6*, *F4/80*, *Nos2*, *Arg1*, *Mmp9*, and *Tnfa* were analyzed by real-time PCR using specific primers. Conjunctival mRNA transcripts of *Cd4*, *Il17a*, and *Ifnγ* were also measured. *Gapdh* was used as an internal control. One-way ANOVA, *p < 0.05, **p < 0.01, ***p < 0.001, and ****p < 0.0001. Data are shown as the mean ± SEM. The experiments in **a**–**d** were repeated three times independently with similar results, and the data of one representative experiment was shown. *LGE* lacrimal gland excision, *DED* dry eye disease

The aqueous tear-deficient DED model that mimics the reduced tear production was recently achieved by lacrimal gland ablation [48]. Thus, we examined the therapeutic potential of 7E in an LGE-induced aqueous tear-deficient DED model. Mice were subjected to extra-orbital lacrimal gland excision bilaterally, and sham was used on the control mice on day 1. Drugs, including PBS, Xiidra, mIgG, and 7E, were topically applied to mice twice daily from day 8. Both the Xiidra- and 7E-treated groups showed reduced damage to the corneal epithelium on days 10 and 14 compared with the PBS- and mIgG-treated control groups (Fig. 6a, b). Tear production tests were performed on days 4, 7, 10, and 14 for each group. The 7E group showed an increase in tear production on days 10 and 14 compared to the mIgG-treated control group (Fig. 6c). However, the Xiidra-treated group showed no increase in tear production on days 10 and 14 compared to the PBS-treated control group (Fig. 6c). A previous study found that extra-orbital LGE promotes the expansion of Th17 cells in the conjunctiva and draining lymph nodes [42]. Thus, the conjunctiva and draining lymph nodes were also harvested to analyze the population of CD4^+^ T cells. The FACS analysis demonstrated that both Xiidra-treated and 7E-treated groups displayed significant decreases in the Th17 cell population compared to the PBS-treated and mIgG-treated groups (Additional file 1: Fig. S13). The expression of several proinflammatory factors, including *Il1b*, *Il6*, *F4/80*, *Nos2*, *Mmp9*, and *Tnfa*, were significantly upregulated in the cornea of PBS-treated and mIgG-treated groups, and the effects were reversed after 7E treatment (Fig. 6d). In addition, the expression of *Cd4* and *Il17a* in the conjunctiva showed similar results (Fig. 6d). However, the expression of *Ifnγ*, which is a marker of Th1 cells, showed a non-significant difference between these groups (Fig. 6d). The ratio of *Bax/Bcl-2* in the conjunctiva and the amounts of apoptotic cells in cornea and conjunctiva were decreased after 7E treatment (Additional file 1: Fig. S14).

### 7E alleviates disease severity in the DS-induced DED model

**Fig. 7:**
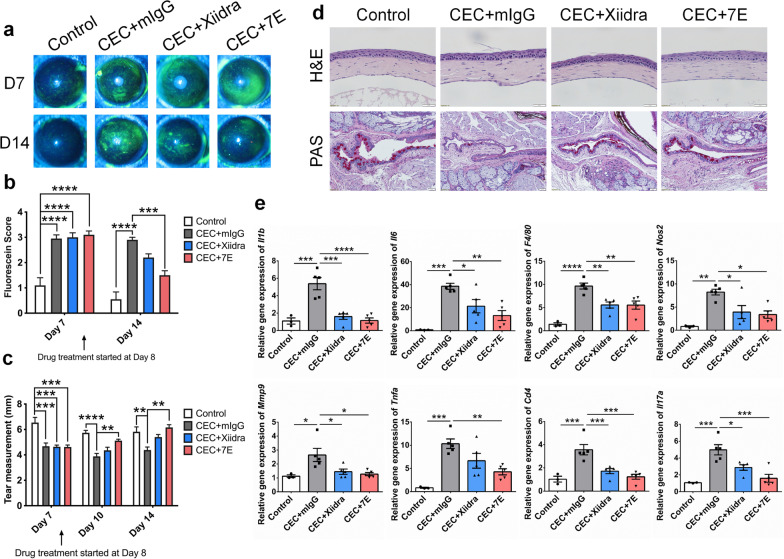
7E ameliorated disease severity in the DS-induced DED animal model. Mice were given a total of four subcutaneous injections of scopolamine in a low humidity-controlled environment chamber for 14 days to induce desiccating stress (DS)-induced DED. Uninduced mice were used as healthy controls (n = 3). Drugs including mIgG, 7E, and Xiidra (each group, n = 5) were administered topically three times a day from day 8. All mice were sacrificed on day 14 and eye tissue was removed. **a** Corneal fluorescein staining was performed to analyze the integrity of the corneal epithelium. Representative images were taken with a cobalt blue filter. **b** The fluorescein scores were blindly evaluated. One-way ANOVA, ***p < 0.001, and ****p < 0.0001. Data are shown as the mean ± SEM. **c** Tear production was measured at the same time of day in the standard environment. One-way ANOVA, ** *p* < 0.01, *** *p* < 0.001, and ****p < 0.0001. Data are shown as the mean ± SEM. **d** H&E staining was performed to observe corneal morphology. Original magnification: ×400. PAS staining was used to visualize the mucin content (red) in the conjunctiva. Nuclei were counterstained with hematoxylin (blue). Original magnification: ×200. **e** The corneal mRNA transcripts of *Il1b*, *Il6*, *F4/80*, *Nos2*, *Mmp9*, and *Tnfa* were analyzed by real-time PCR with specific primers. The conjunctival mRNA transcripts of *Cd4* and *Il17a* were also analyzed. *Gapdh* was used as an internal control. One-way ANOVA, *p < 0.05, **p < 0.01, ***p < 0.001, and ****p < 0.0001. Data are shown as the mean ± SEM. The experiments in **a**–**e** were repeated three times independently with similar results, and the data of one representative experiment was shown. *CEC* controlled environment chamber, *DED* dry eye disease

Mice were housed in a CEC with low humidity (relative humidity < 25%) for 14 days and injected with scopolamine to induce mixed aqueous tear-deficient and evaporative dry eye. Drugs, including mIgG, PBS, Xiidra, and 7E, were topically applied to mice three times daily since day 8. 7E-treated groups showed reduced fluorescein scores on day 14 compared with mIgG-treated control group (Fig. 7a, b). 7E treatment promoted tear production on days 10 and 14 compared to the mIgG-treated control group (Fig. 7c). Xiidra did not significantly increase tear production on days 10 and 14 compared to the mIgG-treated control group (Fig. 7c). 7E effectively maintained cornea epithelium integrity and mucin content (Fig. 7d). CEC induced the expression of several proinflammatory factors, including *Il1b*, *Il6*, *F4/80*, *Nos2*, *Mmp9*, and *Tnfa*, in the cornea (Fig. 7e). The 7E-treated group displayed a significant decrease in the expression of these inflammatory factors in the cornea, and Th17 cells-related factors, including *Cd4* and *Il17a*, were also reduced in the conjunctiva after 7E treatment (Fig. 7e). 7E treatment also reduced *Bax/Bcl-2* ratio and the amounts of apoptotic cells in cornea and conjunctiva (Additional file 1: Fig. S15).

## Discussion

Increasing evidence suggests that inflammation is the core mechanism causing the vicious cycle in DED. Our results demonstrated that the tear levels of IL-20, as well as the proinflammatory cytokines IL-6 and IL-8, were elevated in patients with DED compared with non-DED controls, suggesting the critical role of inflammatory mediators in the pathogenesis of DED. Several animal models have been used to mimic DED conditions in patients. The pathogenesis of DED, however, is multifactorial, and each model has its own limitations and cannot fully reproduce the pathophysiological mechanisms that occur in patients with DED. Therefore, we employed three different mouse models of DED to investigate the pathogenic role of IL-20. The first is the widely used BAC-induced model to study inflammatory and the evaporative type of DED [27]. The second is the LGE-induced DED model by surgical removal of the extra-orbital lacrimal gland to mimic the aqueous tear-deficient with non-Sjögren’s dry eye type [27, 49]. The third is the DED model induced by scopolamine administration and desiccation stress (DS) to mimic mixed evaporative and aqueous tear-deficient DED conditions [27]. The results from our in vivo experiments confirmed the upregulation of IL-20 in the corneas and tears of the DED mice in three different models, suggesting that clinical DED mediated by different etiologies may induce IL-20 production. Furthermore, blockade of IL-20 by the monoclonal antibody 7E ameliorated the disease severity in all of these three models, suggesting that IL-20 mediated a shared pathogenic mechanism in the DED models.

In the corneas, IL-20 was majorly upregulated in the corneal epithelial cells in the three DED mouse models. In vitro, we showed that hyperosmotic stress induced IL-20 expression in cornea epithelial cells, whereas this was blocked by NFAT5 inhibitor KRN5, suggesting that the cellular osmotic regulator NFAT5 contributes to the upregulation of IL-20 under hyperosmotic stress conditions. Further study is necessary to investigate whether NFAT5 directly regulates the transcription of IL-20 by binding to the promoter of *IL20* gene. Since tear hyperosmolarity is one of the key mechanisms to trigger ocular surface inflammation and damage in DED [50, 51], long-term exposure to hyperosmotic stress stimulates corneal epithelial cells to initiate a cascade of inflammatory events, leading to the loss of mucin-producing goblet cells and the activation of immune cells to amplify the inflammatory response [52]. Our results demonstrated that 7E reduced hyperosmotic stress-induced cell apoptosis and proinflammatory cytokines production, which further supports that IL-20 is a potential downstream mediator in the context of hyperosmotic stress-induced corneal inflammation.

Hyperosmotic stress triggers apoptotic cell death by regulating BAX and BCL-2 expression and caspase-3 activation in human corneal cells [53, 54]. Our results showed that IL-20 directly enhanced cell death in corneal epithelial cells while 7E treatment was effective in protecting corneal epithelial HCE-2 cells from hyperosmotic stress- and BAC-induced cell death by regulating BAX and BCL-2. In vivo, treatment with 7E significantly reduced the ratio of *Bax/Bcl2* and the number of apoptotic cells in the cornea and conjunctiva (goblet cell-rich region) in three different DED models. BAC was reported to cause cell death in the cornea and conjunctival cells [55, 56]. Conjunctival epithelial cells are important in secreting the mucin MUC5AC to stabilize the tear layer and moisten the surface of the eye [57], whereas the decreased tear film production due to the loss of conjunctival epithelial cells or altered expression of MUC5AC has been implicated in the pathogenesis of DED [58]. In vivo, 7E treatment prevented the loss of mucin and MUC5AC contents in the BAC-induced model, which suggests that IL-20 upregulation in dry eye conditions may cause loss of corneal epithelial integrity and damage to conjunctival goblet cells to reduce tear production while targeting IL-20 by 7E could provide protective effects to maintain the functions of corneal and conjunctival cells.

In addition to the protective effect of 7E on corneal epithelial cells, targeting IL-20 by 7E reduced hyperosmotic stress-mediated polarization and activation of M1 macrophages in vitro. Our recent study found that IL-20 enhances the polarization of macrophages into proinflammatory M1-type, which supports our results [59]. In our BAC-induced DED mouse model, 7E treatment reduced corneal tissue infiltration of F4/80^+^ macrophages. Interestingly, IL-20 is also detected on the infiltrated F4/80^+^ macrophage population in the DED corneas, suggesting that IL-20 could be an autocrine or paracrine factor to activate macrophages and result in forming a vicious cycle of the inflammatory responses in DED. Macrophages are crucial immune mediators in inflammatory dry eye disease [14, 47, 60] and that macrophage depletion was found to protect against DED in an experimental model by reducing proinflammatory cytokine release [16]. Further study to investigate whether IL-20-mediated macrophage activation is crucial during the DED progression is required.

Increasing evidence indicates that Th17 cells play an important role in DED by upregulating its inducers, including IL-6, IL-23, and IL-17A, in the tears of patients with DED [61]. Additionally, Th17 cell numbers are increased in the ocular surface and in draining lymph nodes in DED animal models [61, 62]. Th17 cells not only cause a loss of cornea integrity by promoting the production of MMP-9 but also induce an autoimmune response by suppressing Treg responses in draining lymph nodes [61]. In our LGE-induced DED model, both 7E and Xiidra treatment reduced the Th17 cell population in the conjunctiva and draining lymph nodes. 7E significantly reduced the expression of *Cd4* and *Il17a* in LGE and DS models. These results indicate that IL-20 could mediate the activation of Th17 and that 7E can effectively block the Th17 cell response in DED.

In our study, the therapeutic efficacy of 7E and Xiidra in mouse DED models was compared. Xiidra, an FDA-approved drug for DED, is the antagonist of the LFA-1 that effectively blocks the interaction between ICAM-1 and LFA-1 and its downstream inflammatory response in DED [20]. Although Xiidra and 7E displayed similar therapeutic effects that protected against BAC-induced over-evaporative DED, 7E showed greater benefits in maintaining tear production than Xiidra in the LGE-induced aqueous tear-deficient DED and DS-induced combined over-evaporative and aqueous tear-deficient DED. Therefore, 7E may be more effective for patients with DED with aqueous tear-deficient or combined over-evaporative and aqueous tear-deficient symptoms. It has been reported that some patients with DED do not respond to Xiidra treatment [63]. Therefore, administration of an anti-IL-20 mAb may represent an alternative strategy for treating this condition, especially for patients with aqueous tear-deficient or combined over-evaporative and aqueous tear-deficient DED. Furthermore, the mechanisms of action seemed to differ between Xiidra and 7E. Xiidra blocks T cell activation by disrupting the interaction between LFA-1 and ICAM-1 [19], while 7E suppresses IL-20-mediated corneal epithelial damage and inhibits macrophage activation to diminish the inflammatory response. Furthermore, because 7E also inhibited the expression of ICAM-1, it may have the same effect as Xiidra on T cell activation thereby providing a broader spectrum of protection against DED.

In clinical settings, several side effects of Xiidra have been reported, including headache, eye irritation, itchy eyes, dysgeusia, sinusitis, and allergic reactions [19, 21]. Therefore, the development of antibody drugs with higher specificity than small molecule drugs and fewer off-target adverse effects may help to overcome this issue [64]. However, there are no effective antibody eye drops on the market for treating DED currently. The anti-IL-20 antibody was well tolerated and showed no safety issues in healthy volunteers and patients with rheumatoid arthritis [65], which demonstrated the safety of anti-IL-20 as a new therapeutic drug for DED. Our findings suggested that the various complex etiologies of DED induce hyperosmotic stress in corneal epithelial cells, resulting in the release of various proinflammatory factors, leading to an inflammatory response, and IL-20 is the key mediator. IL-20 may function as a feed-forward paracrine factor to amplify the inflammatory responses, including promoting the activation of macrophages and inducing cell death, exacerbating the progression of DED, and creating a vicious circle. According to our results, anti-IL-20 antibody (7E) treatment significantly blocked the expression of several proinflammatory cytokines and improved the severity of DED in animal models. Therefore, IL-20 signaling plays an important role in the progression of DED, and inhibition of this pathway may be a potential therapeutic strategy.

Our study has some limitations. Through the collection of clinical samples, we excluded patients with autoimmune diseases-mediated DED, including patients with Sjögren’s syndrome. Therefore, none of the DED groups covered in this study were associated with autoimmune diseases. Whether IL-20 mediates the pathogenic role in Sjögren’s syndrome-associated DED is worthy of further investigation. Also, the number of samples included in this study is still not large enough, which is due to the need for rigorous screening and testing of subjects included in the study and to prevent long-term storage of samples. Additionally, whether the levels of IL-20 in tears can be used as a biomarker for the diagnosis or prognosis of the dry eye still needs the support of more clinical samples in further study.

## Conclusions

Our results demonstrate that IL-20 is a promising therapeutic target in DED: (1) IL-20 is elevated in the tears of patients with DED and upregulated in the tears and cornea of DED mouse models; (2) IL-20 expression is induced under hyperosmotic stress by NFAT5 activation; (3) IL-20 promotes the infiltration and activation of macrophages, leading to an intense inflammatory response; (4) The anti-IL-20 mAb 7E protects corneal epithelial cells from hyperosmotic stress-induced cell death by regulating BAX/BCL-2; (5) Blockade of IL-20 signaling by 7E protects against BAC-induced, LGE-induced, and DS-induced DED in three mouse models. Most importantly, 7E is an antibody and may have fewer non-specific side effects, as compared to the small chemical compound of Xiidra. Collectively, our results demonstrated that IL-20 plays a critical role in DED and that the anti-IL-20 antibody 7E is a potential therapeutic for DED.

## Supplementary Information

**Additional file 1: Table S1.** The demographic data of the people involved in this study. **Table S2.** Details of the analysis of cytokine levels in clinical samples. **Table S3.** Primer pairs used for amplification of mRNA transcripts. **Figure S1.** Schematic illustration of the extra-orbital LGE and histology of the excised extra-orbital lacrimal gland. **Figure S2.** Dynamic observation of the BAC-induced DED mouse model. **Figure S3.** Analysis of proinflammatory cytokines and osmolarity in tears from DED animal models. **Figure S4.** The dynamic changes of the proinflammatory cytokines and osmolarity in tears from DED animal models. **Figure S5.** IL-20 is induced under hyperosmotic stress in the HCE-2 cell line. **Figure S6.** Detection of NFAT5 and IL-20 in the HCE-2 cell line. **Figure S7.** Expression levels of IL-20 and its receptors in HCE-2 cells. **Figure S8.** IL-20 promotes the expression of several proinflammatory factors in the HCE-2 cell line. **Figure S9.** IL-20 promotes cell death of corneal epithelial cells and 7E protects corneal epithelial cells from BAC-induced cell death. **Figure S10.** 7E protects against cell death in the BAC-induced DED model. **Figure S11.** Immunohistochemistry of MUC5AC in BAC-induced DED animal model. **Figure S12.** 7E treatment reduces the infiltration of macrophages into the cornea in the BAC-induced DED animal model. **Figure S13.** 7E causes the decrease of the Th17 population in the draining lymph nodes and conjunctiva from the LGE-induced DED animal model. **Figure S14.** 7E treatment reduced apoptosis in the cornea and conjunctiva from the LGE-induced DED animal model. **Figure S15.** 7E protects cornea and conjunctiva cells from apoptosis in the DS-induced DED animal model.

**Additional file 2.** Original DNA gel and Western blot images.

## Data Availability

The dataset(s) supporting the conclusions of this article is(are) available in the (Mendeley Dat) repository, [Wang, Hsiao-Hsuan (2022), “IL-20&DED”, Mendeley Data, V1, 10.17632/mk327f2ryd.1].

## Abbreviations

BAC: Benzalkonium chloride
CEC: Controlled environment chamber
DED: Dry eye disease
DS: Desiccating stress
mAb: Monoclonal antibody
ICAM-1: Interaction between intercellular adhesion molecule 1
IL: Interleukin
LFA-1: Lymphocyte function-associated antigen 1
LGE: Lacrimal gland excision
MMP-9: Matrix metallopeptidase-9
TNF-α: Tumor necrosis factor-α
