# Supplementary material for: Interleukin-20 is involved in dry eye disease and is a potential therapeutic target
Source: J Biomed Sci. 2022 Jun 9;29:36. doi: 10.1186/s12929-022-00821-2 (PMC9178884; doi:10.1186/s12929-022-00821-2)
Supplement: Supplementary file 1 — Additional file 1: Table S1. The demographic data of the people involved in this study. Table S2. Details of the analysis of cytokine levels in clinical samples. Table S3. Primer pairs used for amplification of mRNA transcripts. Figure S1. Schematic illustration of the extra-orbital LGE and histology of the excised extra-orbital lacrimal gland. Figure S2. Dynamic observation of the BAC-induced DED mouse model. Figure S3. Analysis of proinflammatory cytokines and osmolarity in tears from DED animal models. Figure S4. The dynamic changes of the proinflammatory cytokines and osmolarity in tears from DED animal models. Figure S5. IL-20 is induced under hyperosmotic stress in the HCE-2 cell line. Figure S6. Detection of NFAT5 and IL-20 in the HCE-2 cell line. Figure S7. Expression levels of IL-20 and its receptors in HCE-2 cells. Figure S8. IL-20 promotes the expression of several proinflammatory factors in the HCE-2 cell line. Figure S9. IL-20 promotes cell death of corneal epithelial cells and 7E protects corneal epithelial cells from BAC-induced cell death. Figure S10. 7E protects against cell death in the BAC-induced DED model. Figure S11. Immunohistochemistry of MUC5AC in BAC-induced DED animal model. Figure S12. 7E treatment reduces the infiltration of macrophages into the cornea in the BAC-induced DED animal model. Figure S13. 7E causes the decrease of the Th17 population in the draining lymph nodes and conjunctiva from the LGE-induced DED animal model. Figure S14. 7E treatment reduced apoptosis in the cornea and conjunctiva from the LGE-induced DED animal model. Figure S15. 7E protects cornea and conjunctiva cells from apoptosis in the DS-induced DED animal model. [file 12929_2022_821_MOESM1_ESM.pdf]

Additional file 1

Table. S1 The demographic data of the people involved in this study.

Table. S2 Details of the analysis of cytokine levels in clinical samples.

Table. S3 Primer pairs used for amplification of mRNA transcripts.

Fig. S1 Schematic illustration of the extra-orbital LGE and histology of the excised extra-orbital lacrimal gland.

Fig. S2 Dynamic observation of the BAC-induced DED mouse model.

Fig. S3 Analysis of proinflammatory cytokines and osmolarity in tears from DED animal models.

Fig. S4 The dynamic changes of the proinflammatory cytokines and osmolarity in tears from DED animal models.

Fig. S5 IL-20 is induced under hyperosmotic stress in the HCE-2 cell line.

Fig. S6 Detection of NFAT5 and IL-20 in the HCE-2 cell line.

Fig. S7 Expression levels of IL-20 and its receptors in HCE-2 cells.

Fig. S8 IL-20 promotes the expression of several proinflammatory factors in the HCE-2 cell line.

Fig. S9 IL-20 promotes cell death of corneal epithelial cells and 7E protects corneal epithelial cells from BAC-induced cell death.

Fig. S10 7E protects against cell death in the BAC-induced DED model.

Fig. S11 Immunohistochemistry of MUC5AC in BAC-induced DED animal model.

Fig. S12 7E treatment reduces the infiltration of macrophages into the cornea in the BAC-induced DED animal model.

Fig. S13 7E causes the decrease of the Th17 population in the draining lymph nodes and conjunctiva from the LGE-induced DED animal model.

Fig. S14 7E treatment reduced apoptosis in the cornea and conjunctiva from the LGE-induced DED animal model.

Fig. S15 7E protects cornea and conjunctiva cells from apoptosis in the DS-induced DED animal model.

Table. S1 The demographic data of the people involved in this study.

	non-DED (n=40)	DED (n=40)
Sex (Female %)	75%	70%
Age (year, mean±SEM)	55.20±3.219	60.45±2.275
OSDI score (mean±SEM)	8.3±0.6204	27.03±1.563****
Schirmer's test (>10 mm; %)	80%	0%
Schirmer's test (5-10 mm; %)	20%	15%
Schirmer's test (<5 mm; %)	0%	85%
TBUT (>10 s; %)	100%	0%
TBUT (5-10 s; %)	0%	27.5%
TBUT (<5 s; %)	0%	72.5%

Clinically information including sex, age, Ocular Surface Disease Index (OSDI) score, Schirmer's test, and tear breakup time (TBUT) were provided. Two-tailed unpaired t-test, $p^{****}<0.0001$ compared with non-DED group.

Table. S2 Details of the analysis of cytokine levels in clinical samples.

Cytokine	Non-DED (n=40)	DED (n=40)	*P*-value	Fold increase
IL-20 concentration (pg/ml, mean±SEM)	132.0±30.68	574.3 ± 102.7	0.0007***	4.35
IL-6 concentration (pg/ml, mean±SEM)	81.84±26.11	288.7 ± 71.21	0.0132*	3.52
IL-8 concentration (pg/ml, mean±SEM)	5063±675	8173 ± 652.9	0.0007***	1.61

Cytokine assessment results for non-DED group and DED group. Two-tailed unpaired t-test, *p**<0.05 and *p****<0.001, DED group compared with non-DED group.

Table. S3 Primer pairs used for amplification of mRNA transcripts.

Gene	Forward (5' to 3')	Reverse (5' to 3')
m*Il20*	CCTAAACCACCACCCCTT	CATCACCTGCTCTTGACAGG
m*Il6*	GGCGGATCGGATGTTGTGAT	GGACCCCAGACAATCGGTTG
m*Il1b*	GCAACTGTTCCTGAACTCAACT	ATCTTTTGGGGTCCGTCAACT
m*Mcp1*	AGAGGTCTCGGTTGGGTT	ATCATAACGTTCTGGGCACC
m*Icam1*	AGACTGTGAACACGTGTGACC	ATGTGATCTTTCCCCAGACTC
m*Il10*	GAATGCCTTTAATAAGCTCCA AG	CGTTCACAGAGAAGCTCAGTAA
m*Bax*	TGAAGACAGGGGCCTTTTTG	AATTCGCCGGAGACACTCG
m*Bcl2*	ATGCCTTTGTGGAACTATATGGC	GGTATGCACCCAGAGTGATGC
m*Tnfa*	CCAAATGGCCTCCCTCTCAT	CACTTGGTGGTTTGCTACGA
m*Mmp9*	CTGGACAGCCAGACACTAAAG	CTCGCGGCAAGTCTTCAGAG
m*F4/80*	CATAATCGCTGCTGGTTGAA	GATGAAAATCTGGGCAATGG
m*Nos2*	GTTCTCAGCCCAACAATACAAGA	GTGGACGGGTCGATGTCAC
m*Arg1*	CTCCAAGCCAAAGTCCTTAGAG	AGGAGCTGTCATTAGGGACATC

m*Cd4*	AGGTGATGGGACCTACCTCTC	GGGGCCACCACTTGAACTAC
m*Il17a*	TTTAACTCCCTTGGCGCAAAA	CTTTCCCTCCGCATTGACAC
m*Ifnγ*	ATGAACGCTACACACTGCATC	CCATCCTTTTGCCAGTTCCTC
m*Gapdh*	AGCCTCGTCCCGTAGACA	GATGACAAGCTTCCCATC
hICAM1	GGCTGGAGCTGTTTGAGAAC	TCACACTGACTGAGGCCTTG
hIL-6	CAAGAGTAACATGTGTGA AAGC	CTCCTTAAAGCTGCGCAGAA
hIL-8	TAGCAAAATTGAGGCCAAGG	AGGCACAGTGGAACAAGGAC
hIL-1β	ACTGAAAGCTCTCCACCTC	GAGGTGCTGATGTACCAGTTG
hIL-20	ATGAAAGCCTCTAGTCTTGCCT	GCCCCGTATCTCAGAAAATCC
hIL-20R1	ACAAAGTGTTCCAAATGGGCT	TGGGACCACGTTCTGTTTGAT
hIL-20R2	GGCCACTGTGCCATACAAC	TCTTTGGTGATCTCCATCCCA
hIL-22R1	CACCCCAGACACGGTCTACA	GGCTTGAGGGTAGTGTGCTG
hMMP-9	TGTACCGCTATGGTTACACTCG	GGCAGGGACAGTTGCTTCT
hBAX	TTTGCTTCAGGGTTTCATCC	CAGTTGAAGTTGCCGTCAGA
hBCL-2	GGTGGGGTCATGTGTGTGG	CGGTTCAGGTACTCAGTCATCC

hTNF-α	ATGGCGTGGAGCTGAGAGA	CAATGATCCCAAAGTAGACCTG
hMCP-1	CCCCAGTCACCTGCTGTTAT	TCCTGAACCCACTTCTGCTT
hGAPDH	GTATCGTGGAAGGACTCATG	TCTTCCTCTTGTGCTCTTGC

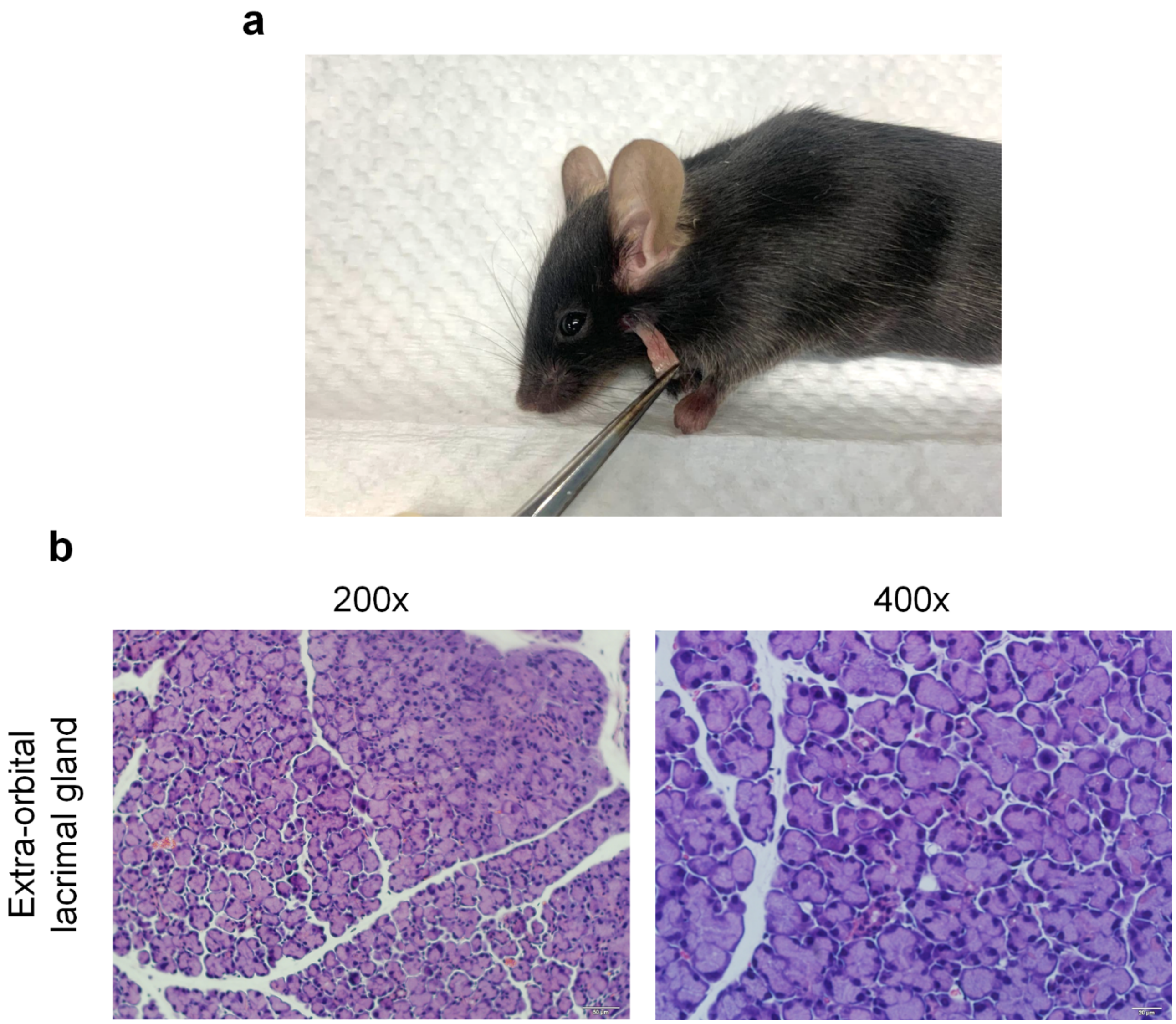

Fig. S1 Schematic illustration of the extra-orbital LGE and histology of the excised extra-orbital lacrimal gland.

a Mice were anesthetized and the extra-orbital lacrimal gland was accessed through an incision made below the ear. **b** H&E stain of the excised extra-orbital lacrimal glands.

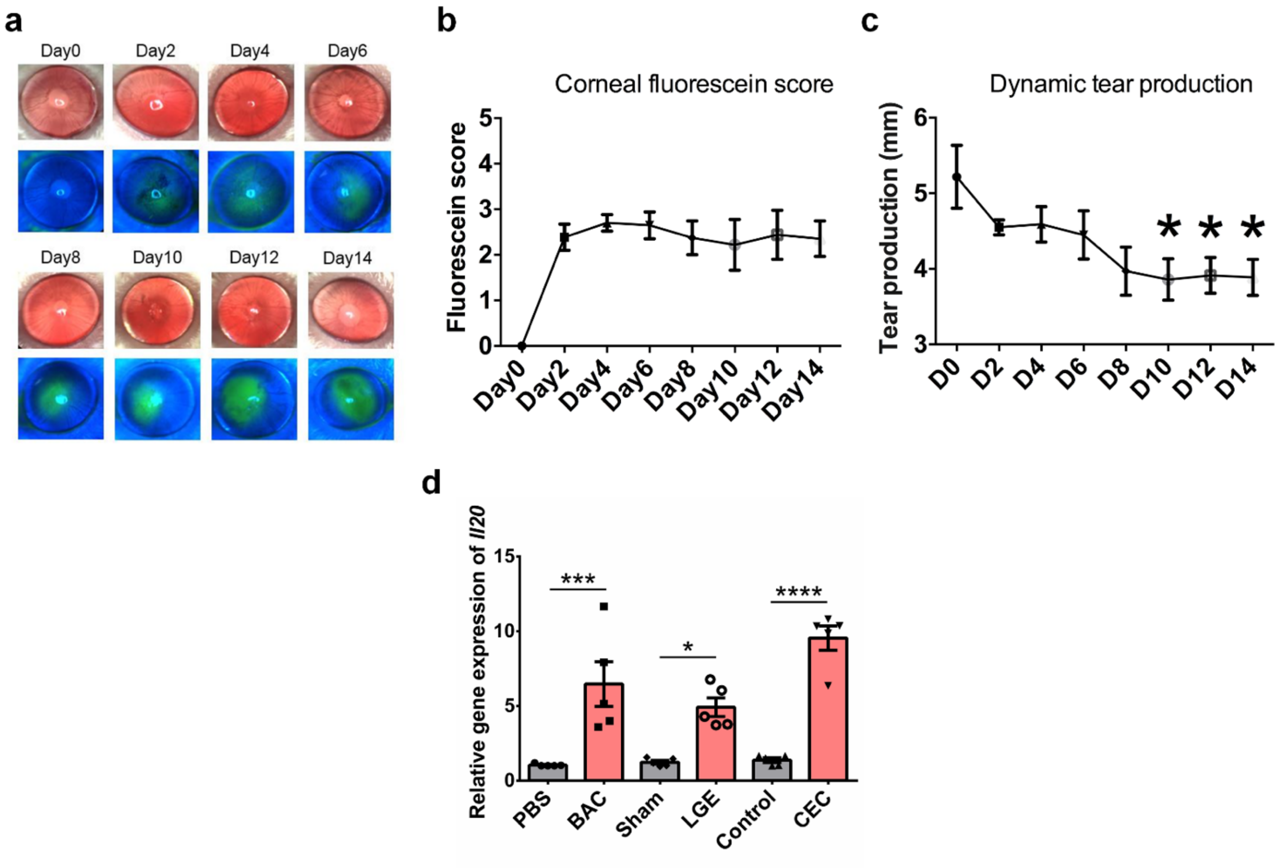

Fig. S2 Dynamic observation of the BAC-induced DED mouse model.

a Mice were topically administrated with BAC twice daily for two weeks and analyzed the clinical DED characteristics every two days (n=5, each group). Corneal fluorescein staining was used to observe the damage of the cornea. **b** The pictures of corneal fluorescein stain were quantified by four researchers by blind tests as fluorescein scores. **c** Tear production (mm) was measured by SMTube Testing. One-way ANOVA, * $p < 0.05$. Data are means ± SEM. **d** Mice were topically administered PBS or BAC twice daily for two weeks to induce DED (for each group, n=5). Bilateral extra-orbital lacrimal gland excision was applied to induce an aqueous tear-deficient DED animal model for two weeks, sham was used for control mice (for each group, n=5). Mice were housed in a CEC with low humidity for 14 days for DS-induced DED; non-induced mice served as healthy controls (for each group, n=5). Mice were sacrificed on day 14 and corneal mRNA transcripts of *Il20* were analyzed using real-time PCR with specific primers. *Gapdh* was used as an internal control. One-way ANOVA, * $p < 0.05$, *** $p < 0.001$, and **** $p < 0.0001$. Data are shown as the mean ± SEM.

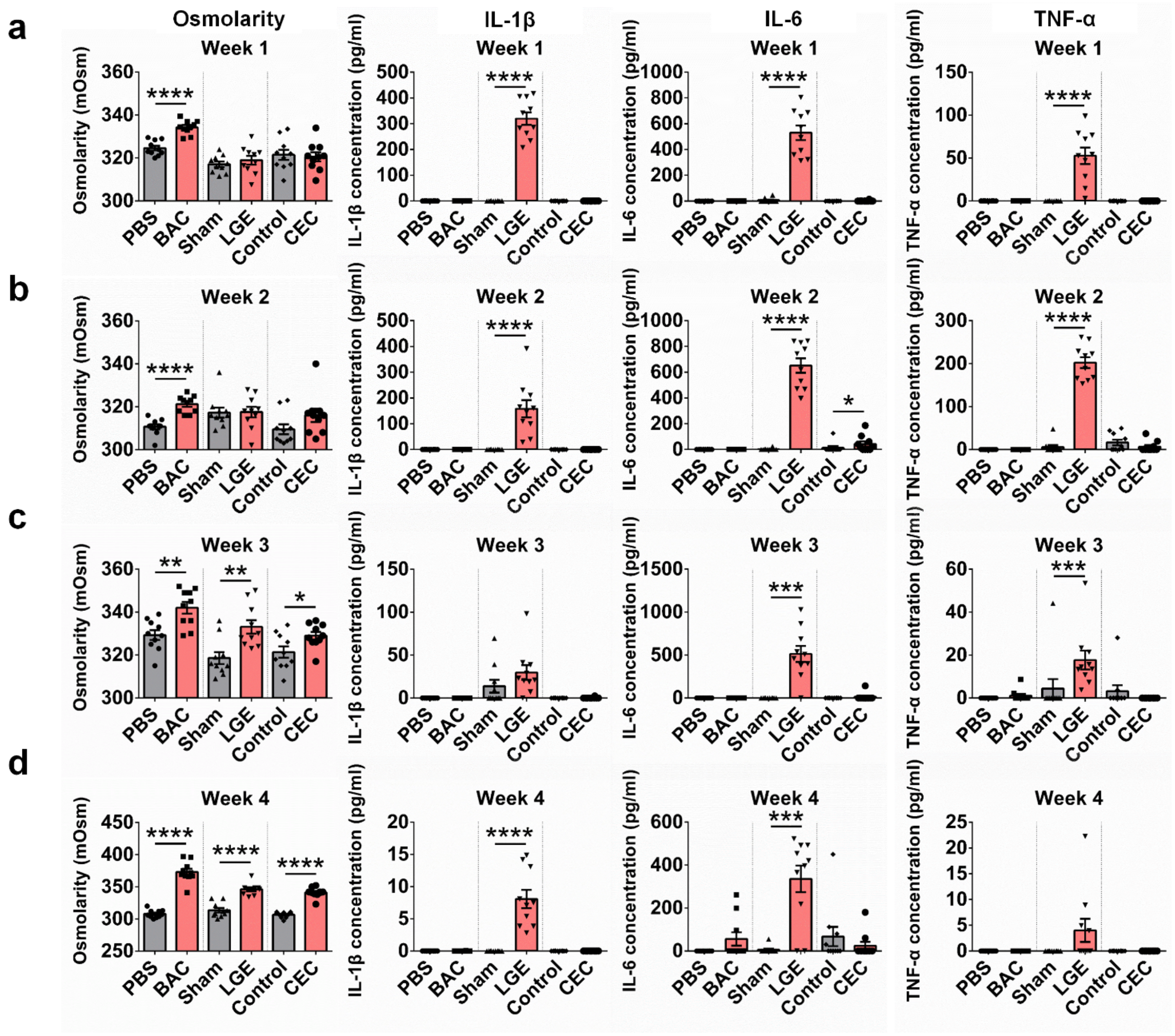

Fig. S3 Analysis of proinflammatory cytokines and osmolarity in tears from DED animal models. **a-d** Mice were topically administered PBS or BAC twice daily for four weeks to induce DED (for each group, n=10). Bilateral extra-orbital LGE was applied to induce an aqueous tear-deficient DED animal model for four weeks, and sham was used for control mice (for each group, n=10). Mice were housed in a CEC with low humidity for 28 days for DS-induced DED; non-induced mice served as healthy controls (for each group, n=10). Mice were harvested tear each week and analyzed for protein levels of IL-1β, IL-6, and TNF-α and osmolarity by ELISA and osmometer. One-way ANOVA, * $p < 0.05$, ** $p < 0.01$, *** $p < 0.001$, and **** $p < 0.0001$. Data are shown as the mean ± SEM.

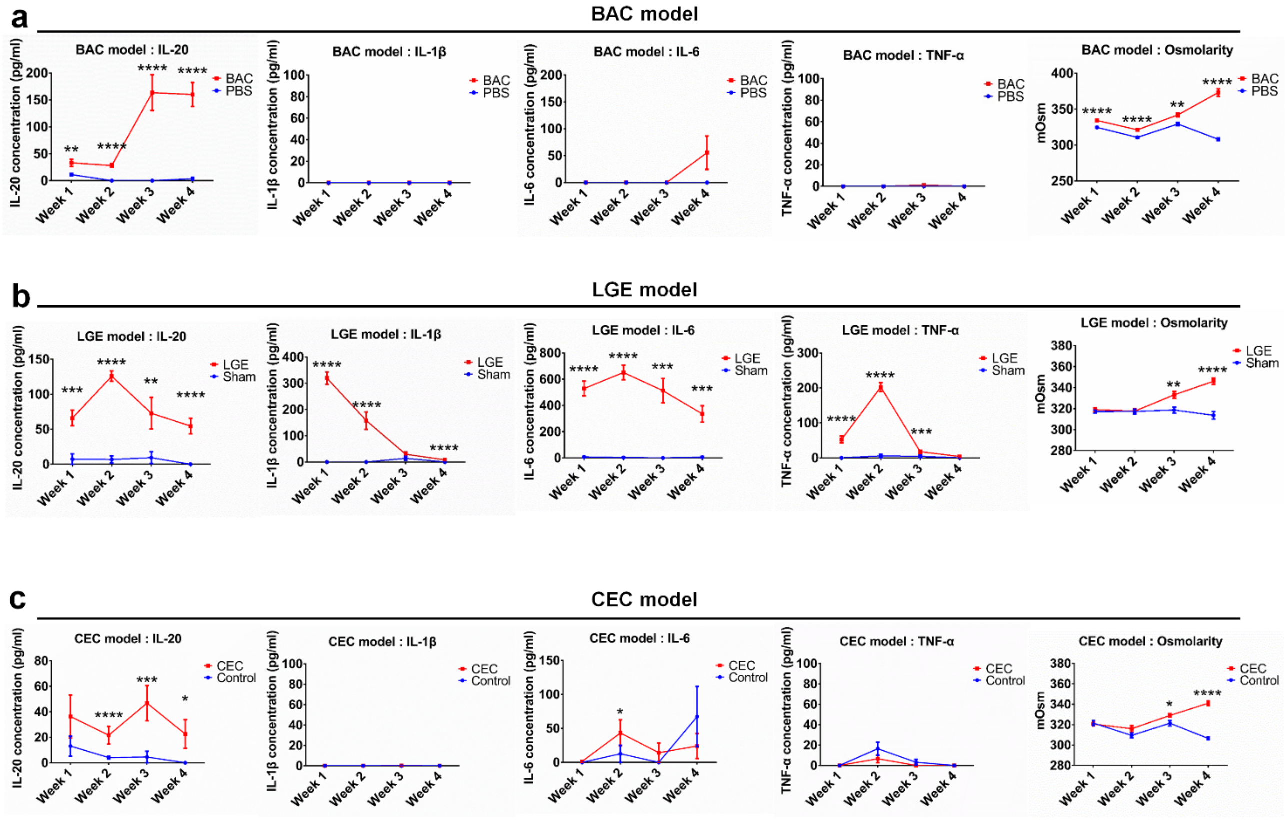

Fig. S4 The dynamic changes of the proinflammatory cytokines and osmolarity in tears from DED animal models.

a-c The dynamic changes of IL-20, IL-1β, IL-6, TNF-α, and osmolarity in tears from DED animal models were analyzed (for each group, n=10). One-way ANOVA, * $p < 0.05$, ** $p < 0.01$, *** $p < 0.001$, and **** $p < 0.0001$ compared with the non-DED control groups. Data are shown as the mean ± SEM.

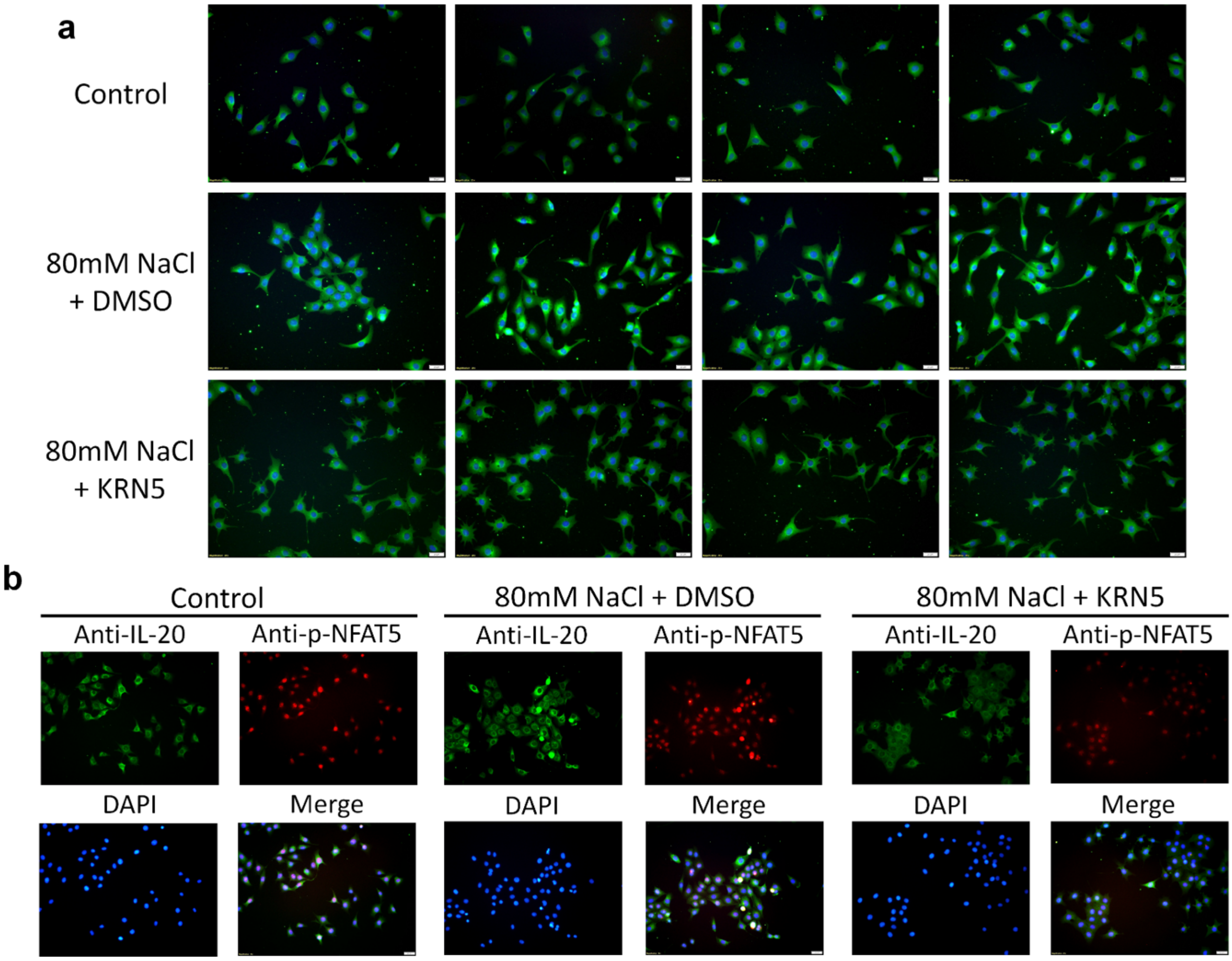

Fig. S5 IL-20 is induced under hyperosmotic stress in the HCE-2 cell line.

a Human corneal epithelial HCE-2 cells were treated with 80 mM NaCl to induce hyperosmotic stress. KRN5 was added to the culture media to inhibit the activation of NFAT5. Cells were incubated for 24 h. Immunofluorescence was applied to stain for the IL-20 (green) and nucleus (blue) in different groups. Original magnification: 200×. **b** Protein levels of IL-20 (green) and phospho-NFAT5 (Ser145) (red) were analyzed by immunofluorescence staining. The nucleus was stained with DAPI (blue). Original magnification: 200×.

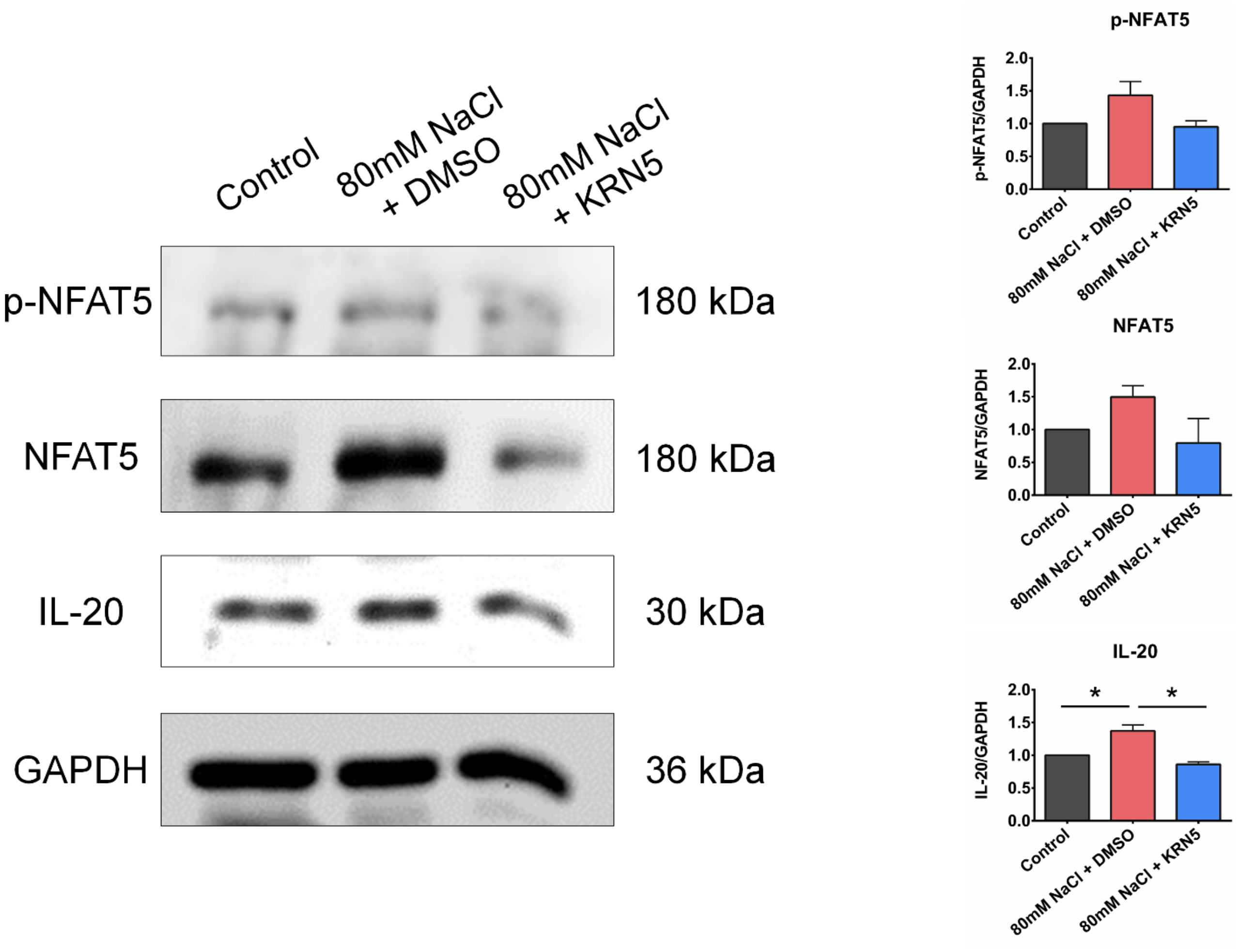

Fig. S6 Detection of NFAT5 and IL-20 in the HCE-2 cell line.

HCE-2 corneal epithelial cells were treated with control or 80 mM NaCl+DMSO or 80 mM NaCl+KRN5 for 24 hours and cell lysates were harvested to analyze the protein levels of NFAT5, phospho-NFAT5 (Ser145), IL-20, and GAPDH (internal control) by western blot. The relative protein levels of NFAT5, phospho-NFAT5 (Ser145), and IL-20 were normalized with GAPDH level and quantified by Image J software (each group, n = 2). One-way ANOVA, * $p < 0.05$. Data are shown as the mean ± SEM.

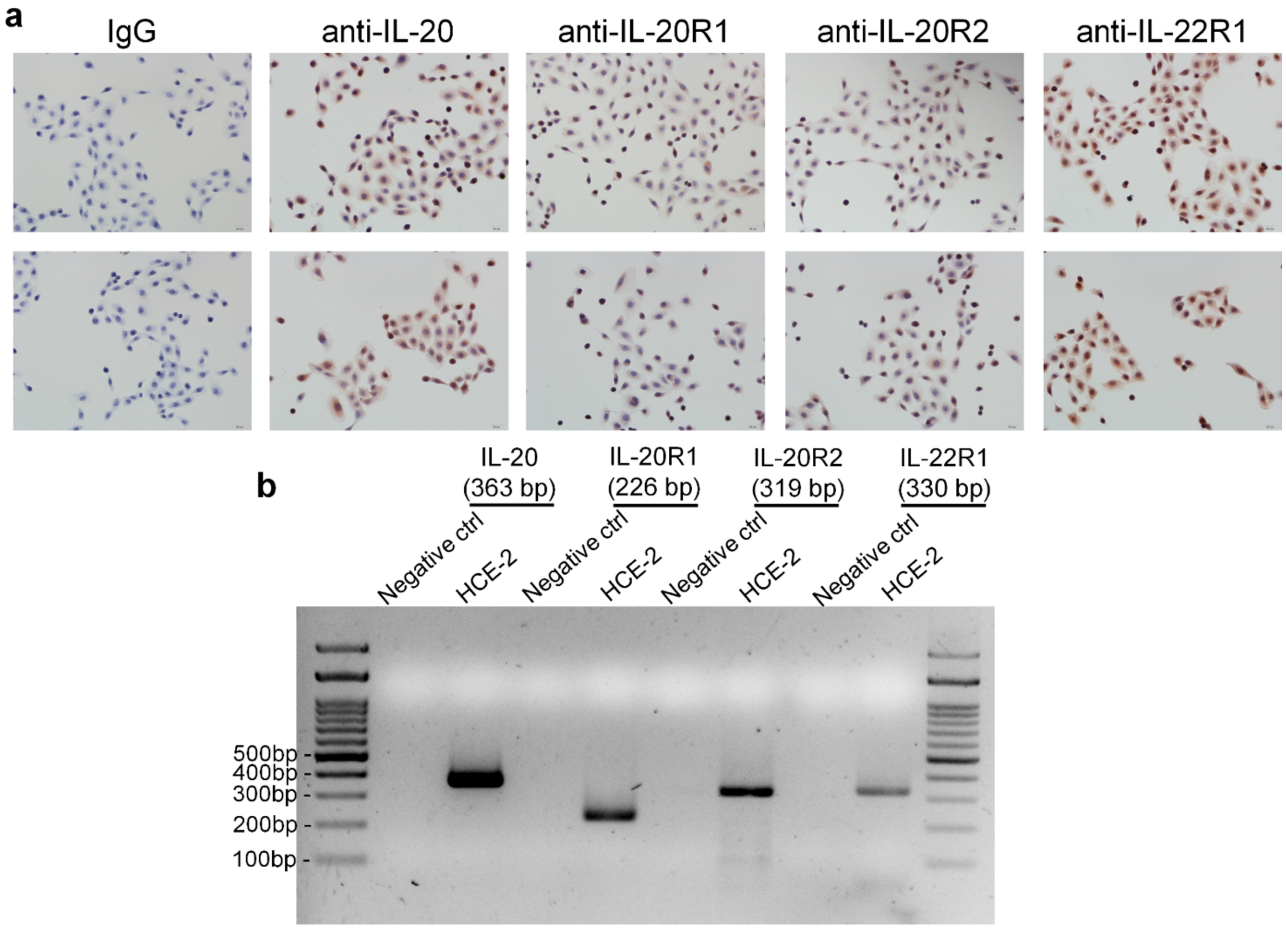

Fig. S7 Expression levels of IL-20 and its receptors in HCE-2 cells.

a Immunocytochemistry staining **b** and RT-PCR were performed to investigate the protein levels and mRNA transcripts of IL-20, IL-20R1, IL-20R2, and IL-22R1 in human corneal epithelial HCE-2 cells.

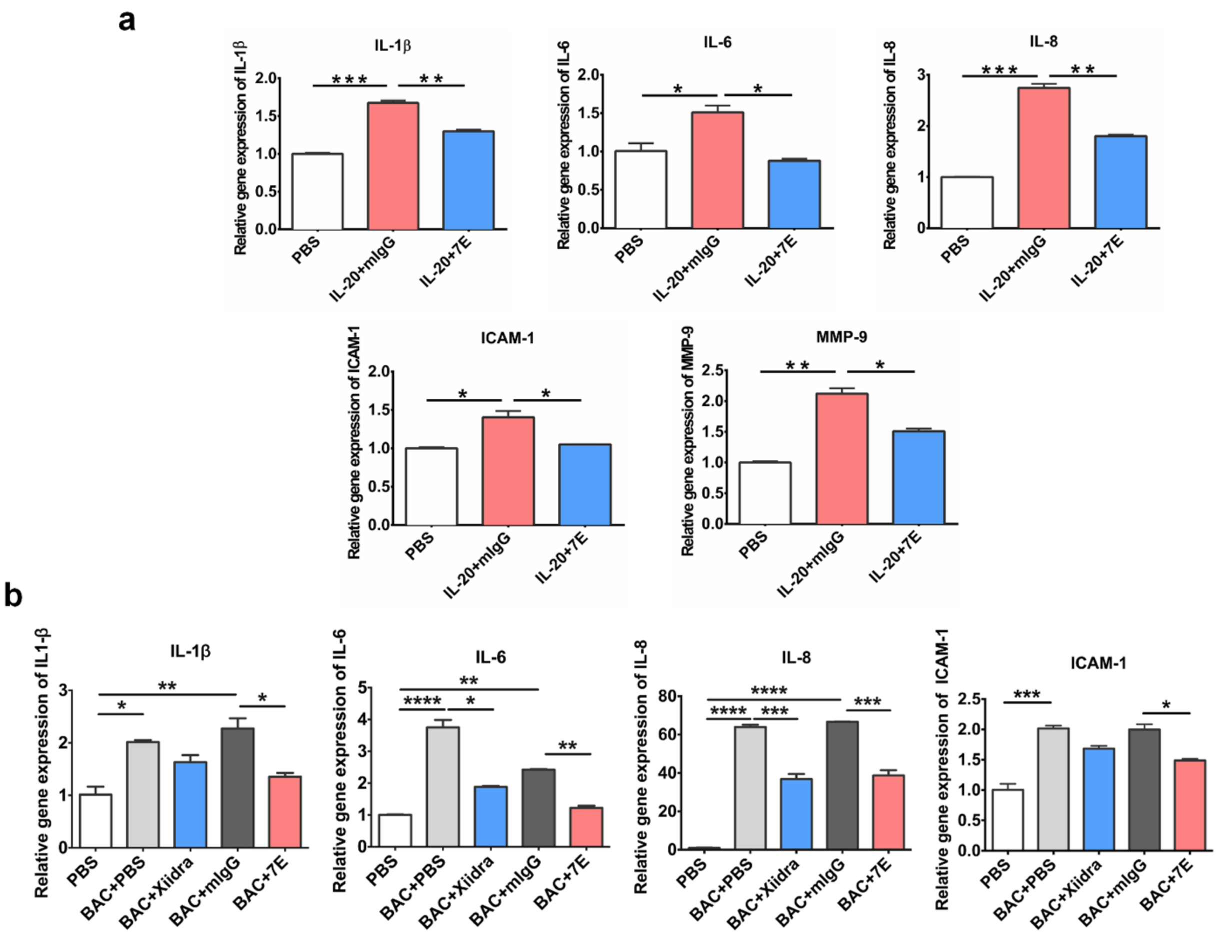

Fig. S8 IL-20 promotes the expression of several proinflammatory factors in the HCE-2 cell line. **a** HCE-2 cells were treated with PBS or IL-20 (400 ng/ml)+mIgG (4 μg/ml) or IL-20+7E (4 μg/ml) for 6h. The corneal mRNA transcripts of IL-1β, IL-6, IL-8, ICAM-1, and MMP-9 were analyzed by real-time PCR with specific primers. GAPDH was used as an internal control. One-way ANOVA, * $p < 0.05$, ** $p < 0.01$, and *** $p < 0.001$. Data are shown as the mean ± SEM. **b** HCE-2 cells were treated with PBS, BAC(0.001%)+PBS, BAC+Xiidra, BAC+mIgG, and BAC+7E for 8 h. Cells were harvested to analyze the gene expression of IL-1β, IL-6, IL-8, and ICAM-1. GAPDH was used as an internal control. One-way ANOVA, * $p < 0.05$, ** $p < 0.01$, *** $p < 0.001$, and **** $p < 0.0001$. Data are means ± SEM.

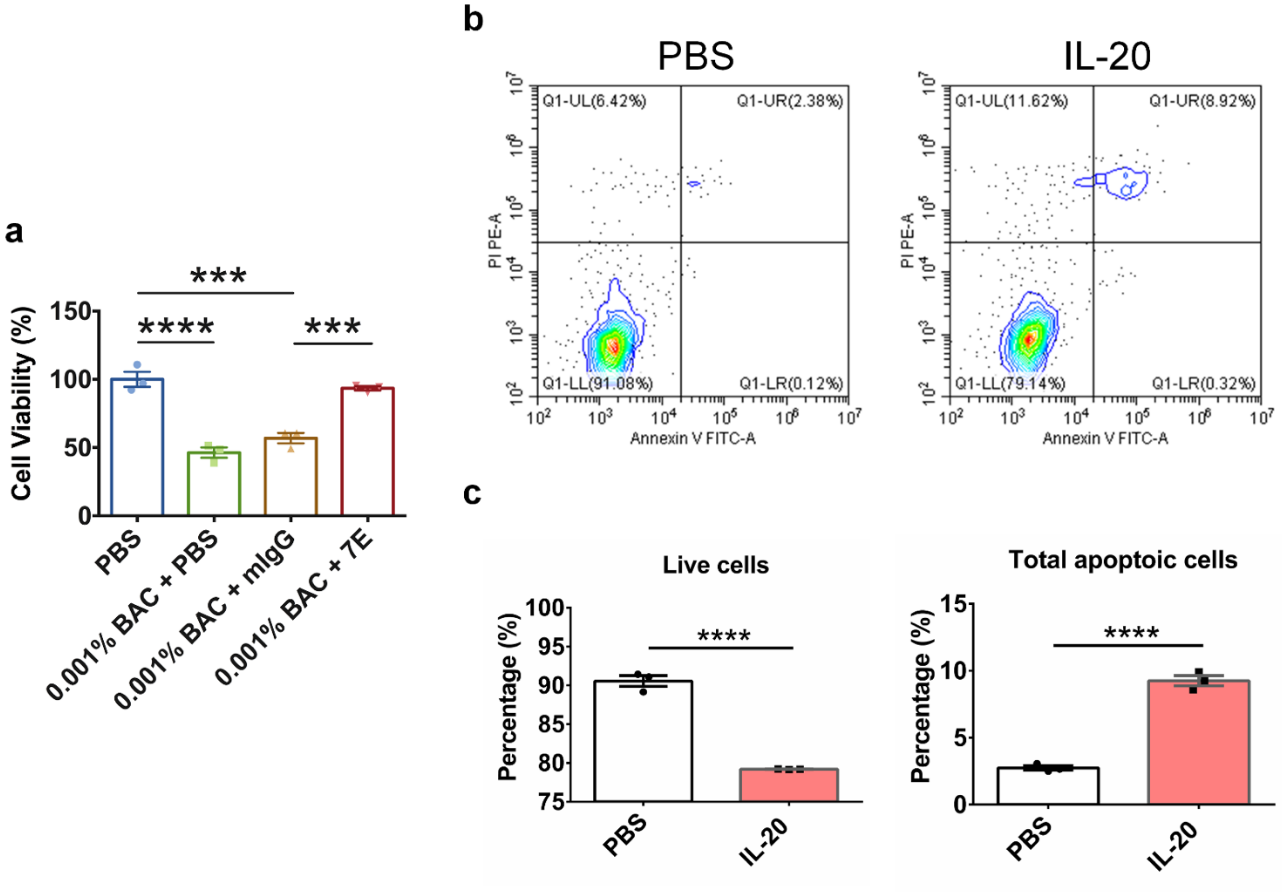

Fig. S9 IL-20 promotes cell death of corneal epithelial cells and 7E protects corneal epithelial cells from BAC-induced cell death.

a Cell viability was determined by a CCK-8 assay to analyze the effects of BAC(0.001%) and 7E (4 μg/ml) on HCE-2 cells. One-way ANOVA, *** $p < 0.001$ and **** $p < 0.0001$. Data are shown as the mean ± SEM. **b** Annexin V/PI staining was applied to investigate the effects of IL-20 on HCE-2 cells. **c** Statistical analysis of the percentages of live and total apoptotic cells according to the results of Annexin V/PI staining. Two-tailed unpaired t-test, **** $p < 0.0001$. Data are shown as the mean ± SEM.

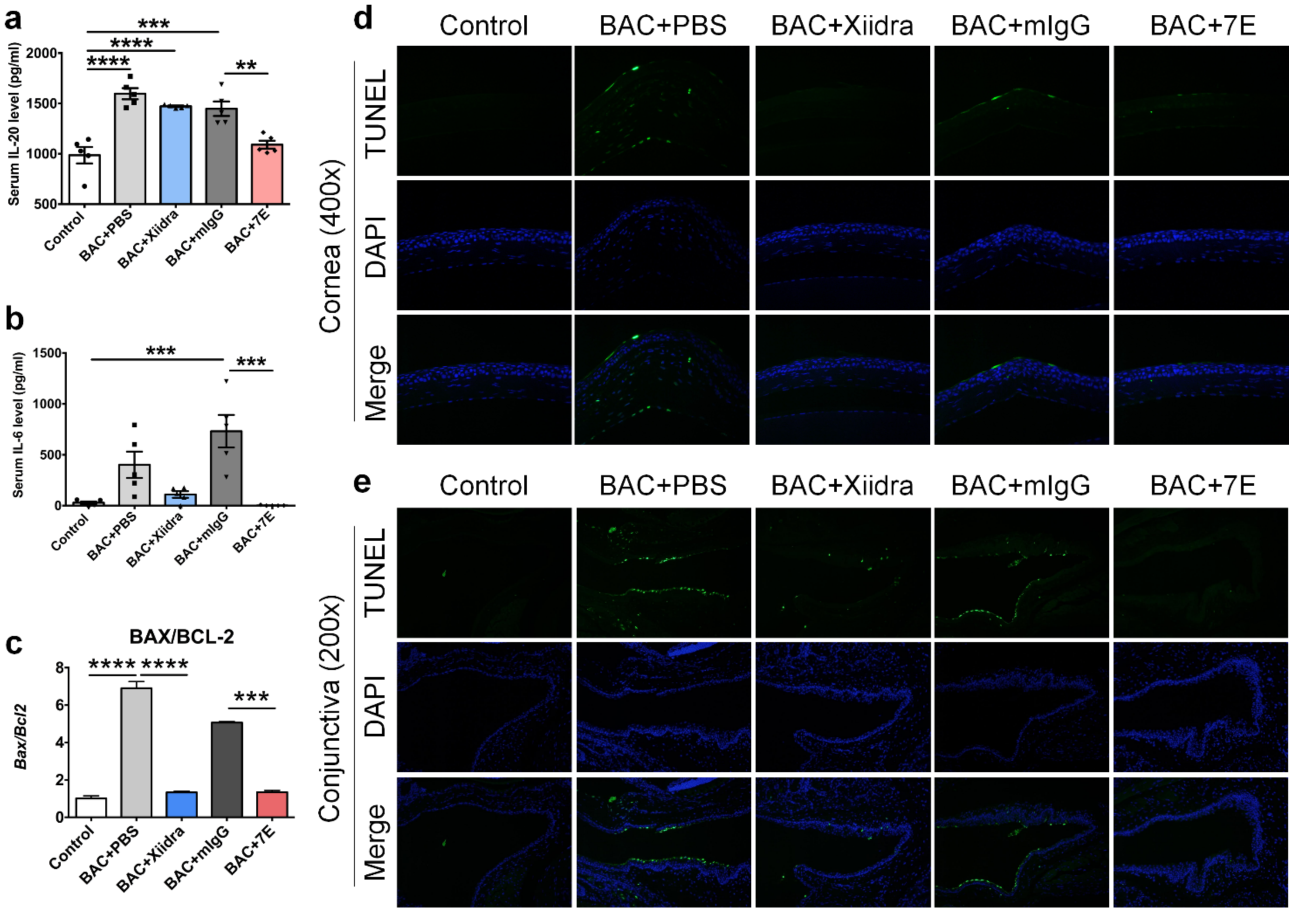

Fig. S10 7E protects against cell death in the BAC-induced DED model.

a-b Mice were sacrificed on day 14, and serum was harvested to detect the levels of IL-20 and IL-6 by ELISA (for each group, n=5). One-way ANOVA, ** $p < 0.01$, *** $p < 0.001$, and **** $p < 0.0001$. Data are shown as the mean ± SEM. **c** BAC-induced DED mice given different treatments were sacrificed on day 14. Corneal transcripts were analyzed to determine the gene expression of *Bax* and *Bcl2* with specific primers. *Gapdh* was used as an internal control. One-way ANOVA, *** $p < 0.001$ and **** $p < 0.0001$. Data are shown as the mean ± SEM. **d-e** TUNEL assay was used to analyze the number of apoptotic cells (green) in the cornea and conjunctival area in different groups. Nuclei were stained with DAPI (blue). Original magnification: 400× and 200×.

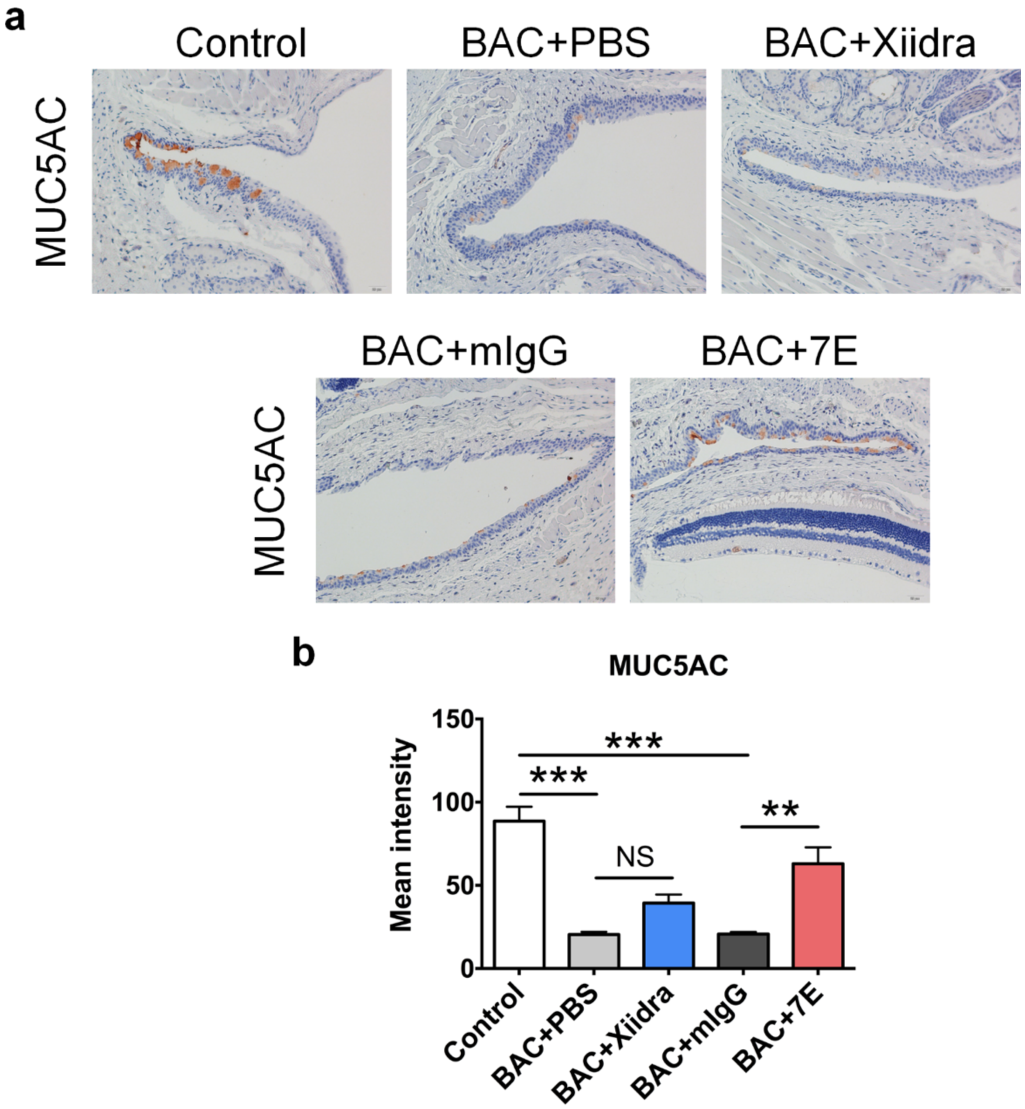

Fig. S11 Immunohistochemistry of MUC5AC in BAC-induced DED animal model.

a Different groups of mice were sacrificed on day 14. The entire eye tissue was isolated for histological analysis. Immunohistochemistry for the MUC5AC (red) and nucleus (blue) in different groups of the dry eye animal model. Original magnification: 200×. **b** The expression level of MUC5AC was quantified by HistoQuest as mean intensity (pixels) (n=3, each group). One-way ANOVA, ** $p < 0.01$ and *** $p < 0.001$. NS represents not significant. Data are means ± SEM.

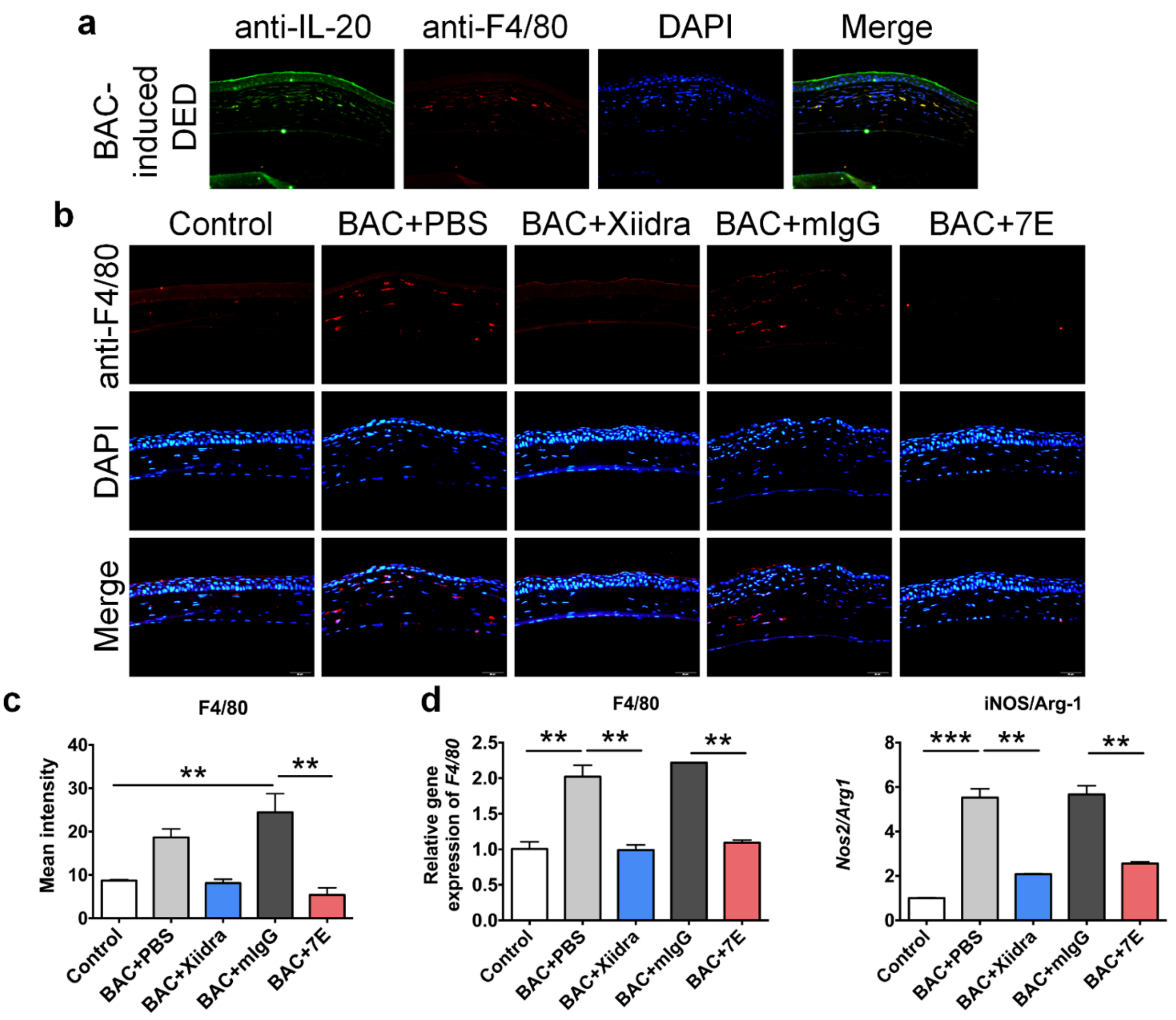

Fig. S12 7E treatment reduces the infiltration of macrophages into the cornea in the BAC-induced DED animal model.

a Mice were topically administered BAC twice daily to induce DED and were sacrificed on day 14. The entire eye tissue was isolated for histological analysis. Immunofluorescence staining for the macrophage marker F4/80 (red), IL-20 (green), and DAPI (blue) in the cornea of a mouse with BAC-induced DED. Co-localization of IL-20 with F4/80 is shown in yellow in the merged image. Original magnification: 400×. **b** Immunofluorescence staining for the macrophage marker F4/80 (red) and DAPI (blue) in different groups of the dry eye animal model. Original magnification: 400×. **c** The expression level of F4/80 was quantified by HistoQuest and is presented as mean intensity (pixels).

One-way ANOVA, ** $p < 0.01$. Data are shown as the mean ± SEM. **d** The corneal mRNA transcripts of *F4/80* (macrophage marker), *Inos* (M1-type macrophage marker), and *Arg1* (M2-type macrophage marker) were analyzed by real-time PCR with specific primers. *Gapdh* was used as an internal control. One-way ANOVA, ** $p < 0.01$ and *** $p < 0.001$. Data are shown as the mean ± SEM.

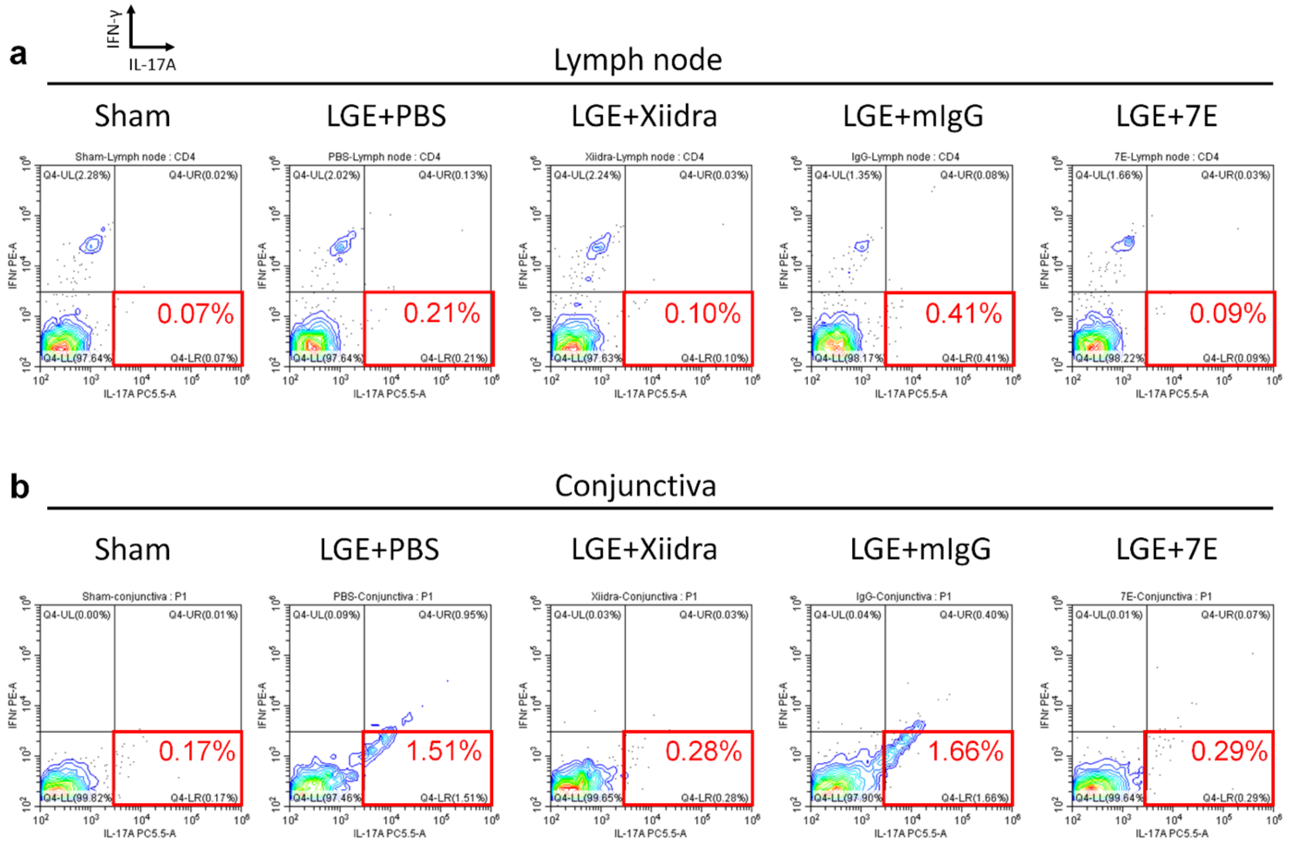

Fig. S13 7E causes the decrease of the Th17 population in the draining lymph nodes and conjunctiva from the LGE-induced DED animal model.

a-b Mice were done with bilateral extra-orbital LGE to the induced aqueous tear-deficient DED for two weeks, different drugs were topical administrated to mice twice a day since day 8 (each group, n = 4). Draining lymph nodes or conjunctiva tissues from four mice in the same group were pooled and analyzed together by FACS. Cells were stained with CD4, IL-17A, and IFN-γ to analyze the Th1 and Th17 cell populations.

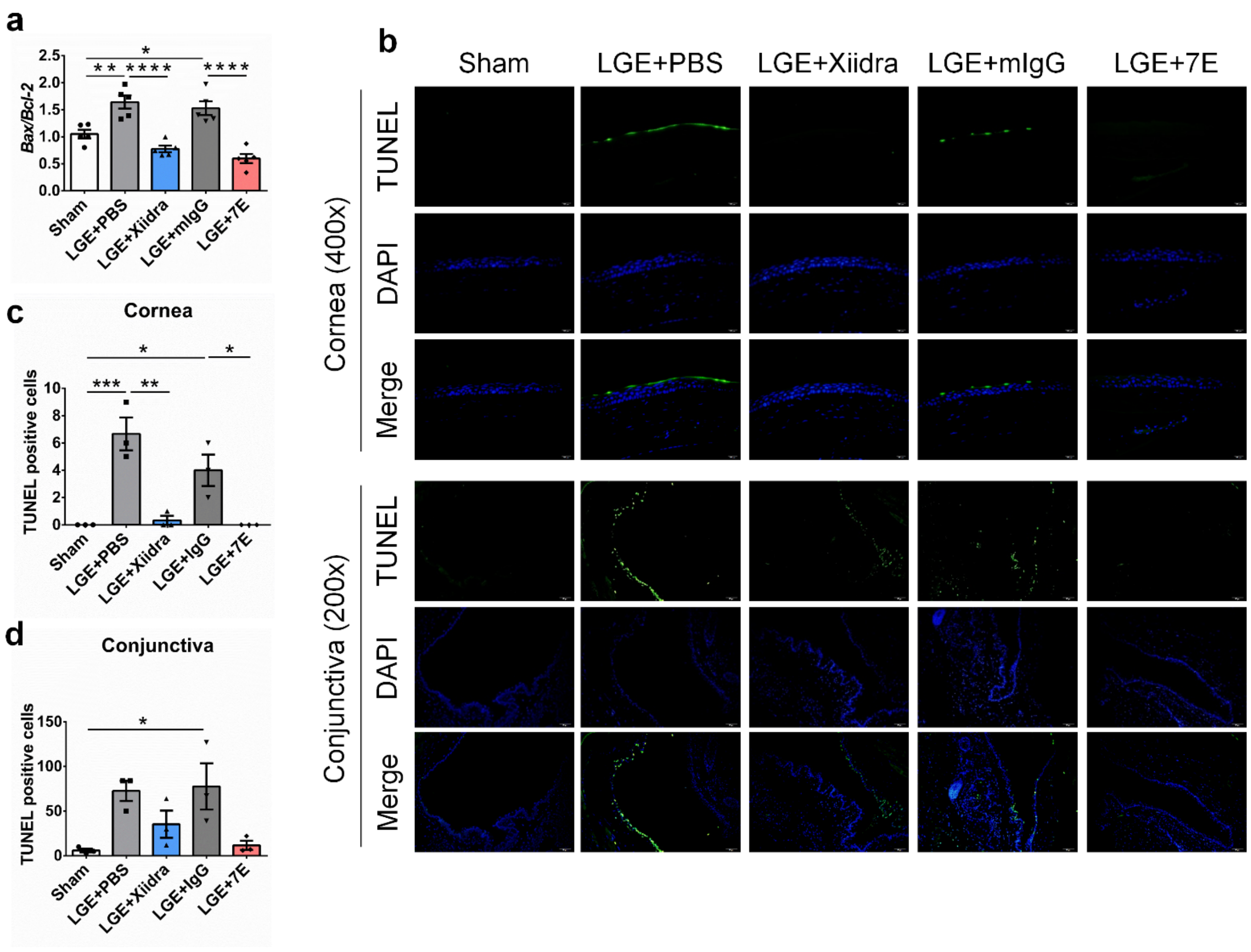

Fig. S14 7E treatment reduced apoptosis in the cornea and conjunctiva from the LGE-induced DED animal model.

a Aqueous tear-deficient DED animal model was induced by extra-orbital LGE on day 1, and mice were treated with drugs, including PBS, Xiidra, mIgG, 7E starting on day 8, and all mice were sacrificed on day 14. Conjunctiva transcripts were analyzed to determine the gene expression of *Bax* and *Bcl2* with specific primers (each group, n = 5). *Gapdh* was used as an internal control. One-way ANOVA, * $p < 0.05$, ** $p < 0.01$, and **** $p < 0.0001$. Data are shown as the mean ± SEM. **b** TUNEL assay was used to detect apoptotic cells (green) in the cornea and conjunctival area in different groups. Nuclei were stained with DAPI (blue). Original magnification: 400× and 200×. **c-d** The amounts of the TUNEL-positive cells were quantified by Image J software (each group, n = 3). One-way ANOVA, * $p < 0.05$, ** $p < 0.01$, and *** $p < 0.001$. Data are shown as the mean ± SEM.

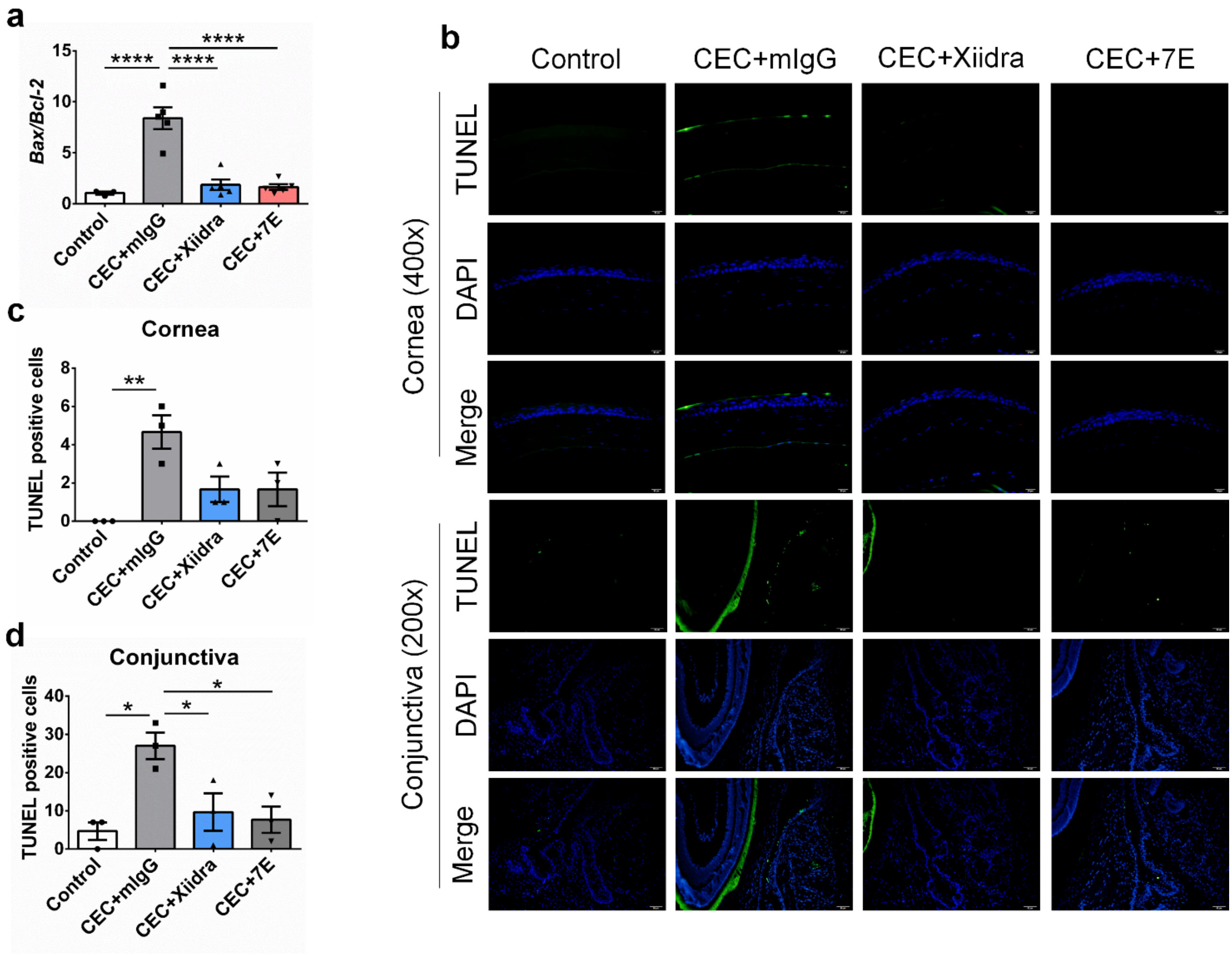

Fig. S15 7E protects cornea and conjunctiva cells from apoptosis in the DS-induced DED animal model.

a Mice were given subcutaneous injections of scopolamine in a low humidity-controlled environment chamber to induce desiccating stress (DS)-induced DED. Uninduced mice were used as healthy controls (n = 3). Drugs including mIgG, 7E, and Xiidra (each group, n = 5) were administered topically three times a day from day 8 and mice were sacrificed on day 14. Conjunctiva transcripts were analyzed to determine the gene expression of *Bax* and *Bcl2* with specific primers. *Gapdh* was used as an internal control. One-way ANOVA, **** $p < 0.0001$. Data are shown as the mean ± SEM. **b** TUNEL assay was used to detect apoptotic cells (green) in the cornea and conjunctiva. Nuclei were stained with DAPI (blue). Original magnification: 400× and 200×. **c-d** The number of the apoptotic

cells was quantified by Image J software (each group, n = 3). One-way ANOVA, * $p < 0.05$ and ** $p < 0.01$. Data are shown as the mean ± SEM.
