# Supplementary material for: Interleukin-20 is involved in dry eye disease and is a potential therapeutic target
Source: J Biomed Sci. 2022 Jun 9;29:36. doi: 10.1186/s12929-022-00821-2 (PMC9178884; doi:10.1186/s12929-022-00821-2)

Additional file 2 (Original DNA gel and Western blot images)

1. Full blot for **Fig. S6 Detection of NFAT5 and IL-20 in the HCE-2 cell line.**

   The images on the right are chemiluminescence images taken in the dark box.

   The pictures on the left are the original membrane picture taken under white light.

(1) Anti-phosho-NFAT5

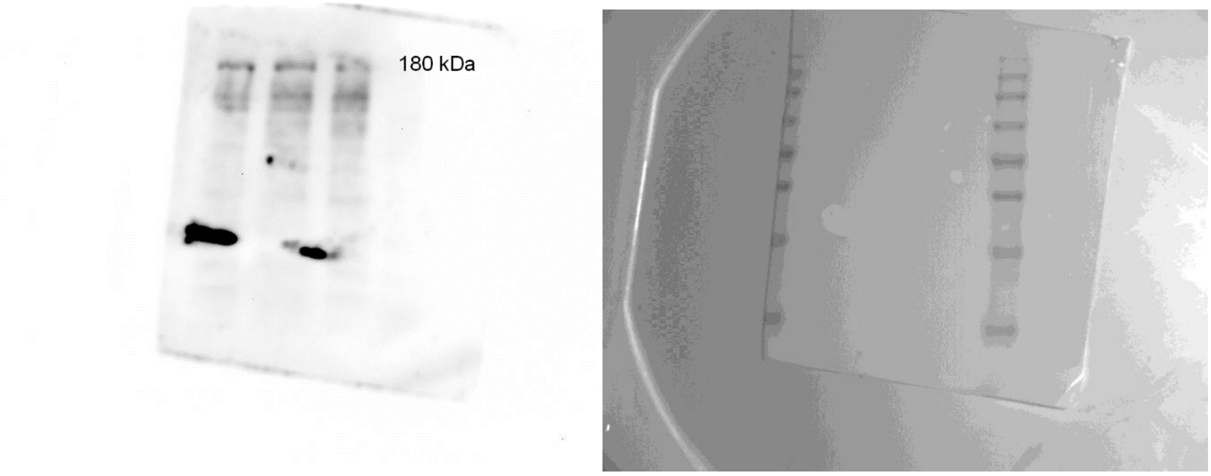

(2) Anti- NFAT5

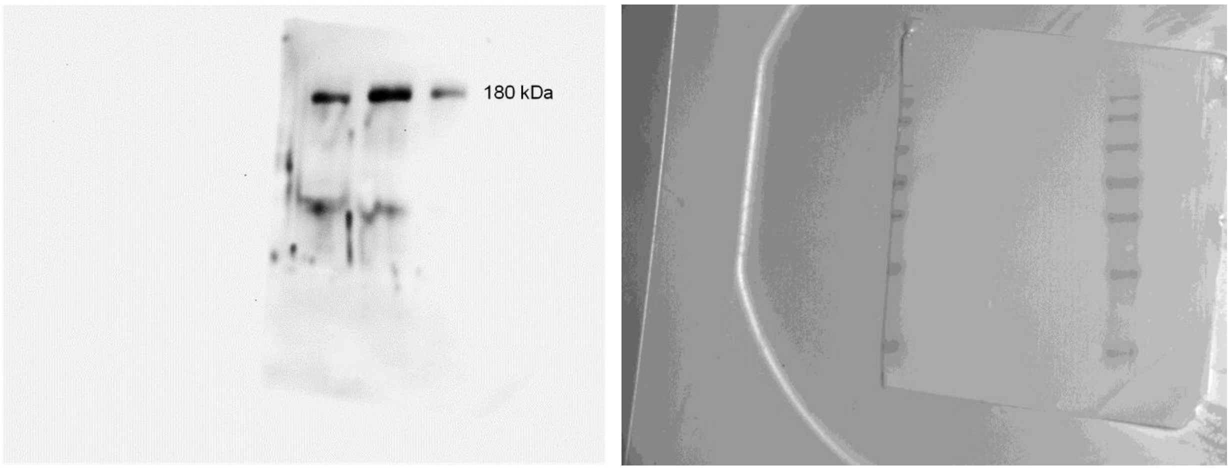

(3) Anti-IL-20

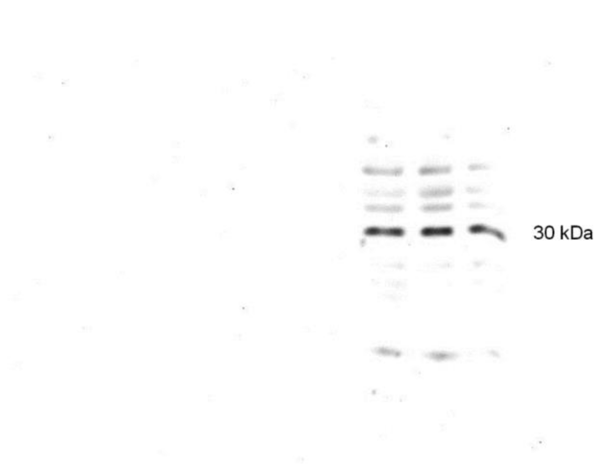

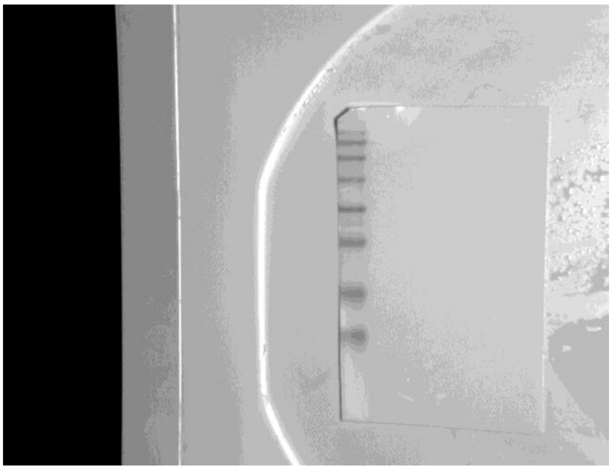

(4) Anti-GAPDH

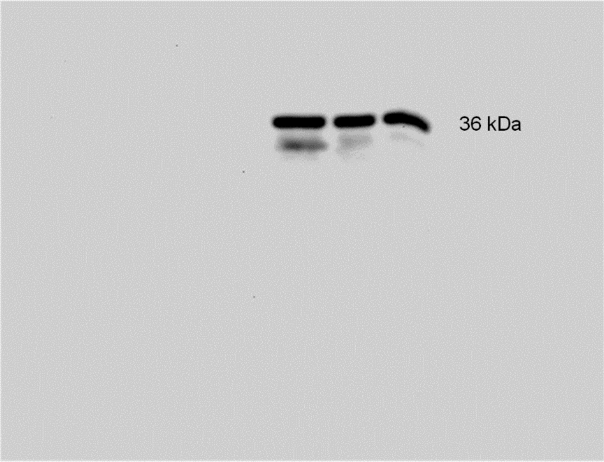

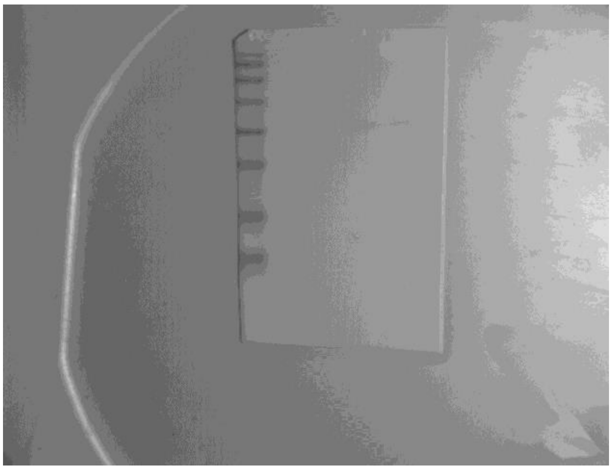

2. DNA gel for **Fig. S7b Expression levels of IL-20 and its receptors in HCE-2 cells.**

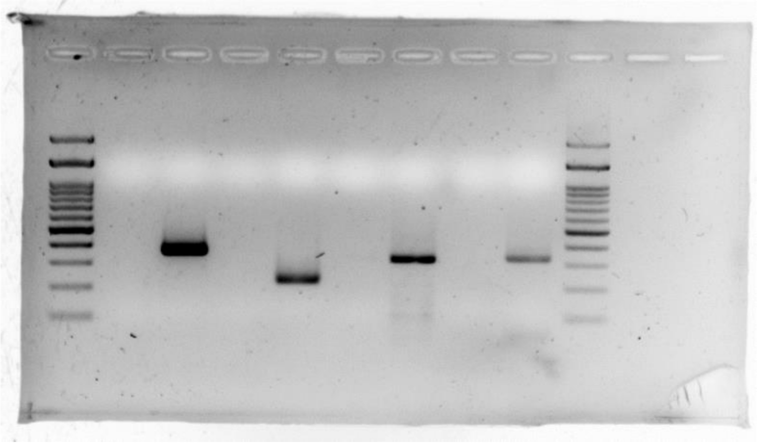

Supplement: Supplementary file 2 — Additional file 2. Original DNA gel and Western blot images. [file 12929_2022_821_MOESM2_ESM.pdf]
